# NF-**κ**B–driven lymphangiogenesis affects kidney function via a VEGFR-3–mediated pathway

**DOI:** 10.1172/jci.insight.198992

**Published:** 2026-01-22

**Authors:** Arin L. Melkonian, Amie M. Traylor, Anna A. Zmijewska, Kyle H. Moore, Gelare Ghajar-Rahimi, Stephanie Esman, Yanlin Jiang, Hani Jang, Babak J. Mehrara, Timmy C. Lee, James F. George, Anupam Agarwal

**Affiliations:** 1 Division of Nephrology, Department of Medicine;; 2 Department of Biomedical Engineering; and; 3 Division of Cardiothoracic Surgery, Department of Surgery, University of Alabama at Birmingham, Birmingham, Alabama, USA.; 4 Plastic and Reconstructive Surgery Service, Department of Surgery, Memorial Sloan Kettering Cancer Center, New York, New York, USA.; 5 Birmingham Veterans Affairs Medical Center, Birmingham, Alabama, USA.

**Keywords:** Cell biology, Nephrology, Lymph

## Abstract

The lymphatic system maintains fluid homeostasis and orchestrates immune cell trafficking throughout tissues. While extensively studied in cancer and lymphedema, its role in nonlymphoid organs, particularly the kidney, remains an emerging area of investigation. Previous research established molecular connections among NF-κB, VEGFR-3, and PROX-1 in regulating lymphatic growth during inflammation, and studies using global knockout mice revealed that the NF-κB1 subunit (p50) influences lymphatic vessel density. However, the role of RelA — a key component of the canonical NF-κB heterodimer — in regulating lymphatic growth and kidney function following acute kidney injury (AKI) remains unexplored. Using an inducible, predominantly lymphatic endothelial cell-specific RelA-knockout mouse model, we demonstrated that RelA expression in VEGFR-3^+^ cells is essential for VEGFR-3–driven lymphangiogenesis following AKI. Knockout mice exhibited substantially worse kidney function, altered histological features, impaired VEGFR-3–dependent lymphangiogenesis, and dysregulated immune cell trafficking compared with WT mice. Compensatory upregulation of PROX-1 and podoplanin occurred despite decreased VEGFR-3 and LYVE-1 total protein expression, suggesting complex regulatory mechanisms. Our findings suggest that RelA is a critical sensor for inflammation and regulator of protective lymphangiogenesis following kidney injury and provide insights into potential therapeutic targets for improved kidney injury outcomes.

## Abstract 2

Our work demonstrates that NF-kB is a critical sensor for inflammation and a key regulator of lymphangiogenesis following acute kidney injury.

## Graphical abstract

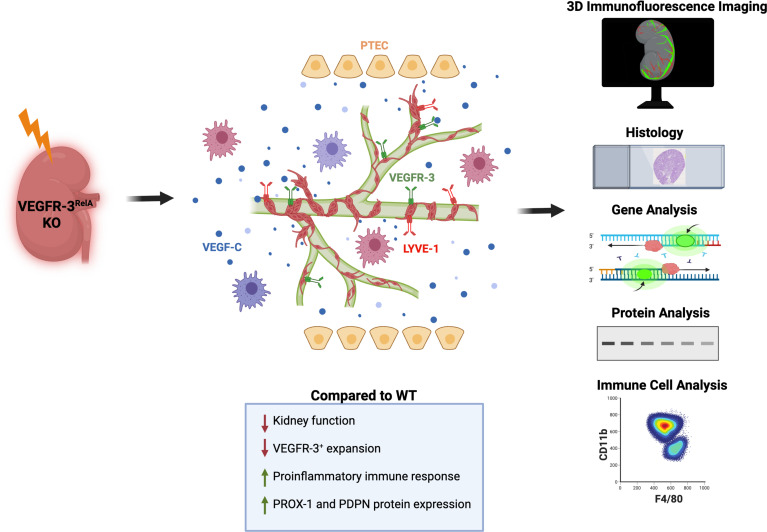

## Introduction

Acute kidney injury (AKI) is one of the leading causes of death in hospitalized patients, and it poses significant challenges to healthcare systems (1, 2). While extensive research has characterized many aspects of AKI pathophysiology, the contribution of the lymphatic system — crucial for maintaining fluid homeostasis, facilitating tissue repair, and trafficking immune cells — remains insufficiently explored in kidney diseases, including AKI and chronic kidney disease (CKD) (3, 4).

The role of lymphangiogenesis (LA), which refers to the formation and development of new lymphatic vessels, has previously been studied in the context of both AKI and CKD. This process is initiated during acute and chronic inflammation when prolymphangiogenic factors, primarily VEGF-C, which is secreted by macrophages and proximal tubules, activate VEGF receptor-3 (VEGFR-3) expressed on the surface of lymphatic endothelial cells (LECs) (5–7).

In response to inflammation, LA helps mitigate kidney injury; when LA is inhibited through VEGFR-3 blockade, kidney function worsens in a cisplatin-induced AKI model and is associated with higher expression levels of NF-κB expression in the kidney (8). Importantly, NF-κB serves as a master transcriptional regulator of inflammatory responses across multiple organ systems. This essential transcription factor is expressed in diverse kidney cell populations — macrophages, lymphocytes, and glomerular, tubular, and endothelial cells — with notably elevated basal expression in LECs throughout multiple organs (9–11).

Despite this evidence, a critical knowledge gap remains regarding which NF-κB pathway components drive LA in the kidney. NF-κB operates through two distinct pathways: canonical (involving p65/RelA and p50/NF-κB1 heterodimer) and noncanonical (involving p100 phosphorylation and nuclear translocation of RelB and p52). The p50/p65 heterodimer is the most abundant NF-κB signaling complex and is highly expressed in most cell types, emphasizing the importance of both subunits working in concert (12, 13). Although studies using global p50-knockout mice have shown decreased lymphatic vessel density in multiple organs, kidney lymphatic density remained unchanged, suggesting possible compensatory mechanisms through noncanonical or other canonical NF-κB pathways (14, 15). While p50 can function as a transcriptional repressor when homodimerized, only p65 contains a C-terminal transactivation domain responsible for gene expression (12). The knockout of p65 itself, the critical transactivation partner of p50, has not yet been explored in the context of lymphatics. Owing to the embryonic lethality of a RelA (p65) knockout, exploring its function in lymphatic development and maturation has historically proven challenging (15, 16).

To address this knowledge gap, we developed a transgenic mouse model with an inducible Cre-Lox system that selectively deletes RelA in VEGFR-3–expressing cells. We chose to target VEGFR-3–expressing cells specifically because there is established evidence of direct crosstalk between NF-κB and VEGFR-3. Mechanistically, prospero homeobox 1 (PROX-1), a canonical lymphatic marker, undergoes activation following NF-κB induction during inflammation. Together, NF-κB and PROX-1 synergistically activate VEGFR-3 in LECs (17). This approach enables temporal control of deletion, circumventing the embryonic lethality observed in conventional knockout mouse models, while allowing investigation of the role of RelA in LA in adult mice. Additionally, RelA has been implicated in kidney injury and contributes to its pathogenesis, which further underscores the importance of NF-κB signaling in AKI (18, 19). Through diverse experimental approaches spanning both in vitro and in vivo lymphatic models, we demonstrated that the knockout of RelA in VEGFR-3^+^ cells (a) exacerbates kidney injury, (b) alters lymphatic transcriptional and protein profiles, (c) promotes compensatory LA in VEGFR-3^–^ LECs, and (d) enhances the acute immune response.

This work elucidates the critical relationship between RelA and LA in the kidney during states of acute inflammation, an area that has remained insufficiently characterized to our knowledge until now. By targeting and enhancing RelA-dependent LA, we could develop new and promising therapeutic strategies to treat AKI and improve patient outcomes.

## Results

### Hypoxia-mediated inflammation induces activation of NF-κB and alters lymphatic expression in human LECs.

As a proof of concept to demonstrate that hypoxia activates NF-κB, human LECs (hdLECs) were subjected to hypoxia (1% O_2_) for several time points: 0 (normoxic), 1, 2, 4, 6, and 16 hours. Hypoxia-inducible factor 1-α (HIF1α), phosphorylated NF-κB (p65 subunit, serine 536 phosphorylation site), and total NF-κB (p65) were probed via Western blot (Supplemental Figure 1A; supplemental material available online with this article; https://doi.org/10.1172/jci.insight.198992DS1). The fold changes of p-p65 (normalized to total p65) and HIF1α (Supplemental Figure 1B) were quantified. Peak p-p65 expression occurred at 16 hours, while HIF1α peaked at 4 hours. HIF1α served as a positive control to confirm successful hypoxic conditions.

To corroborate that hypoxia not only induces p65 protein expression, but also alters lymphatic marker transcript expression, hdLECs were subjected to hypoxia for 30 minutes, 6 hours, and 24 hours. Changes in *Hif1a* transcripts were detected as early as 6 hours, while *Flt4, Pdpn,* and *Lyve-1* were significantly reduced by 24 hours, suggesting that hypoxia significantly altered lymphatic gene expression in a homogeneous population of LECs (Supplemental Figure 1C).

### Knockdown of p50 and p65 subunits alters lymphatic marker expression in hdLECs.

hdLECs were transfected with siRNAs targeting the p50 and p65 subunits of NF-κB for 24 hours. Knockdown efficiency was determined for both p50 and p65 siRNAs, revealing an approximately 80%–90% reduction in expression, as measured by qPCR and RNA analysis (Supplemental Figure 2A).

Knockdown of *p50* significantly increased expression of *Prox-1*, *Flt4*, and *Tnfsf15,* while *p65* silencing led to marked upregulation of *Lyve-1, Pdpn,* and *Flt4* compared with negative scramble controls (Supplemental Figure 2B). These differential expression patterns indicate that both NF-κB subunits play distinct but essential roles in regulating baseline lymphatic marker RNA expression in hdLECs.

### Experimental design and characterization of an inducible, VEGFR-3–specific RelA-knockout mouse model.

**Figure 1:**
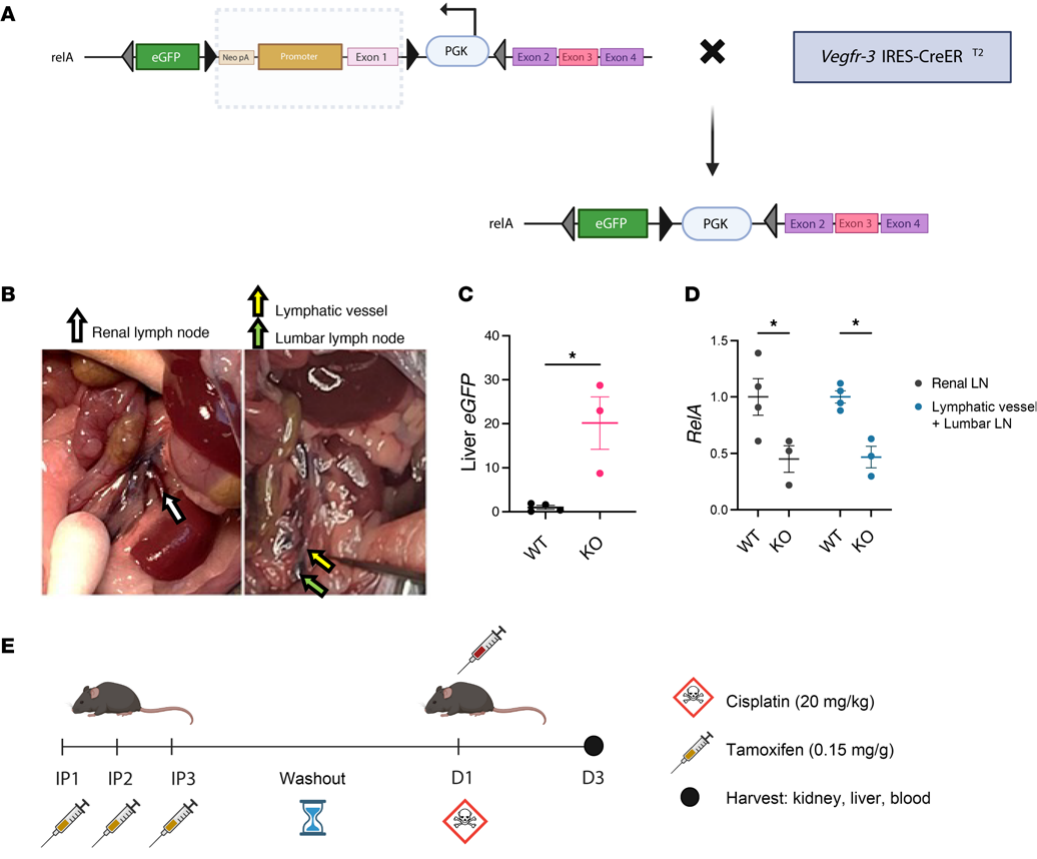
Mouse model, characterization, and experimental design. (**A**) WT mice were crossbred with VEGFR-3 Cre-expressing mice to generate Flt^4Cr^exRel^Aflox^ transgenic animals. Upon successful Cre-mediated recombination, exon 1 of the RelA gene, the neomycin resistance cassette, and promoter region were excised, resulting in subsequent GFP expression. The dotted box represents the components deleted upon recombination. Black and gray triangles denote loxP and frt sites, respectively. WT and its recombined form were adapted from Heise et al. (46). (**B**) Visualization of renal lymph nodes (white arrow) and lymphatic vessels (yellow arrow) with associated lumbar lymph nodes (green arrow) following 5% Evan’s blue dye injection via the footpad, prior to surgical resection. (**C**) Hepatic *eGFP* RNA expression analysis relative to that of WT mice, presented as fold change, and normalized to *Gapdh* confirming successful gene knockout efficiency in tamoxifen-treated knockout mice (*N* = 3) compared with tamoxifen-treated WT controls (*N* = 4). (**D**) Real-time quantitative PCR validation of target gene knockout using primers spanning from exon 1 to exon 3. The observed reduction in RNA expression indicates successful exon 1 excision following Cre-mediated recombination. *RelA* transcript levels were normalized to *Gapdh* expression and are presented as fold change relative to control WT mice (WT, *N* = 4; KO, *N* = 3). (**E**) Experimental timeline depicting sequential tamoxifen and cisplatin treatments, with tissue collection occurring 3 days after high-dose cisplatin (20 mg/kg) or saline administration. The washout period was 10 to 11 days. PGK, phosphoglycerate kinase promoter; Neo pA, neomycin resistance gene-polyadenylation; WT, RelA^fl/fl^; KO, VEGFR-3^RelA–/–^. *P < 0.0332. **C** is unpaired *t* test (2 tailed). **D** is 2-way ANOVA.

For initial baseline characterization (i.e., without injury), Flt4^Cre^xRelA^fl/fl^ transgenic and RelA^fl/fl^ (WT) mice were injected intraperitoneally with tamoxifen for 3 consecutive days to induce Cre-mediated recombination. Successful recombination was confirmed by the expression of GFP following the excision of exon 1 of the RelA gene (Figure 1A). Renal lymph nodes and collecting lymphatic vessels attached to the lumbar lymph nodes were visualized and carefully excised following Evan’s Blue perfusion, while liver tissue samples were also separately collected (Figure 1B). Quantitative PCR analysis revealed significantly elevated *eGFP* expression in liver samples (Figure 1C) and reduced exon 1–specific *RelA* expression in lymph nodes and lymphatic vessels (Figure 1D) of knockout mice compared with WT controls, confirming successful gene knockout. A more robust sample size, including several cohorts treated with tamoxifen, was screened for *eGFP* RNA expression in the liver to confidently confirm knockout (Supplemental Figure 3E).

Following characterization and a washout period, Flt4^Cre^xRelA^fl/fl^ and WT mice were administered either saline (control) or cisplatin (20 mg/kg) via intraperitoneal injection to induce AKI on day 1. At day 3, kidney function was assessed, and kidneys, liver, and blood were collected for analysis (Figure 1E).

### Kidney function assessment of RelA-knockout and floxed control mice.

Interestingly, despite implementing a 10- to 11-day washout period that exceeded the time required for complete systemic clearance of tamoxifen, we observed significant protective effects against kidney injury in tamoxifen-treated mice. This protection was evidenced by marked differences in serum creatinine levels and glomerular filtration rate (GFR) measurements between WT mice receiving cisplatin with either tamoxifen or vehicle control (corn oil) (Supplemental Figure 3, B and C). To ensure experimental rigor, we used tamoxifen-treated WT mice as controls.

To confirm that transgenic Flt4xRelA mice and WT mice had comparable baseline GFRs following tamoxifen administration, measurements were taken prior to injury (Supplemental Figure 3D).

**Figure 2:**
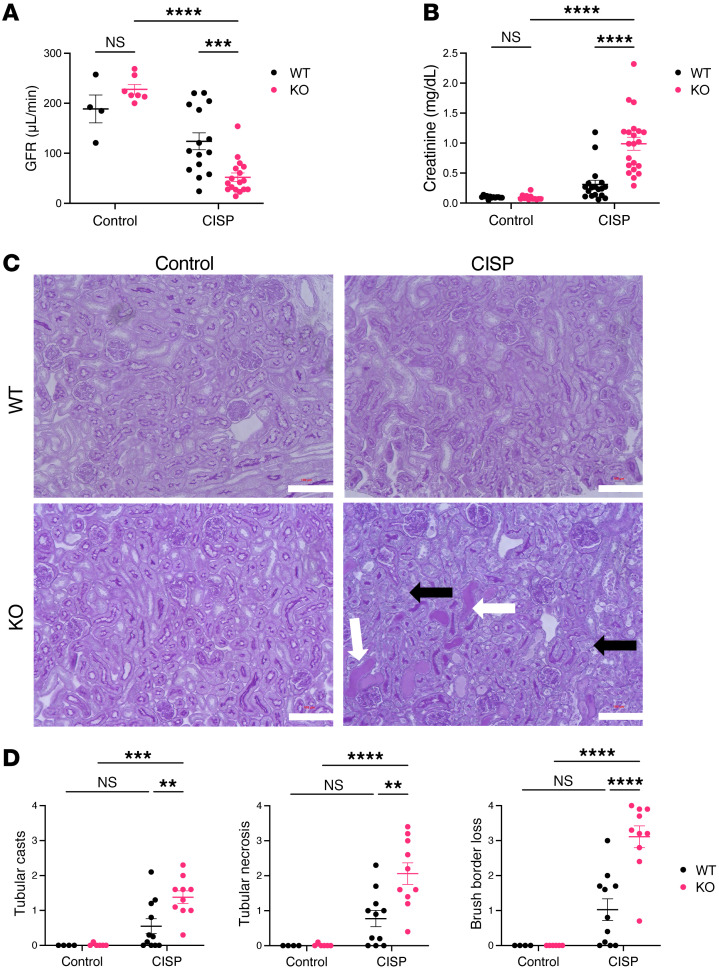
Kidney function and histopathological evaluation of VEGFR-3–specific RelA-deficient mice and floxed controls. (**A**) Glomerular filtration rate (GFR) in control and cisplatin-treated knockout and WT mice (WT control, *N* = 4; KO control, *N* = 7; WT cisplatin, *N* = 15; KO cisplatin, *N* = 17). (**B**) Serum creatinine measurements in control and cisplatin-treated knockout and WT mice. Kidney function analysis included data from at least 4 independent knockout cohorts and 3 WT control cohorts (WT control, *N* = 13; KO control, *N* = 12; WT cisplatin, *N* = 18; KO cisplatin, *N* = 21). (**C**) Representative images of PAS-stained kidney sections from the cortices in knockout and WT mice. Black arrows indicate loss of brush border; white arrows indicate cortical casts. Scale bar: 100 μm. (**D**) Tubular casts, tubular necrosis, and brush border loss were scored in a blinded fashion from 0 (low injury) to 4 (high injury) using an area-based system. Sample sizes (WT control, *N* = 4; KO control, *N* = 6; WT cisplatin, *N* = 11; KO cisplatin, *N* = 10). Statistical significance (*P* < 0.05) was determined using 2-way ANOVA. **P < 0.0021, ***P < 0.002, ****P < 0.0001. WT, RelA^fl/fl^; KO, VEGFR-3^RelA–/–^.

Following cisplatin administration, RelA-knockout mice demonstrated not only a pronounced decline in GFR compared with their baseline measurements, but also significantly lower absolute GFR values compared with cisplatin-treated WT controls (~2.3 fold) (Figure 2A). Supporting these GFR findings, knockout mice exhibited substantially elevated serum creatinine levels (~3.5 fold) compared with WT counterparts, indicating enhanced susceptibility to AKI (Figure 2B).

### Histologic evaluation of kidney injury in RelA-knockout and floxed control mice.

Control (saline-treated) knockout and WT mice showed no visible tubular damage or brush border loss. However, cisplatin-treated knockout mice exhibited clear brush border loss and increased tubular casts (Figure 2C). To quantify visual observations, a nephrologist was blinded to the experimental conditions and scored 3 parameters: (a) tubular casts, (b) tubular necrosis, and (c) loss of brush border by assigning a number 0–4 (0, no injury; 4, severe injury). Tubular necrosis was defined by distorted nuclei, increases in luminal space within tubules, and spindle-shaped morphology. Loss of brush border was determined by the loss of deep purple staining of the brush border. Cisplatin-injured knockout mice demonstrated significantly higher scores for all parameters compared with WT controls (Figure 2D).

### Immune response in RelA-knockout and floxed control mice following injury.

**Figure 3:**
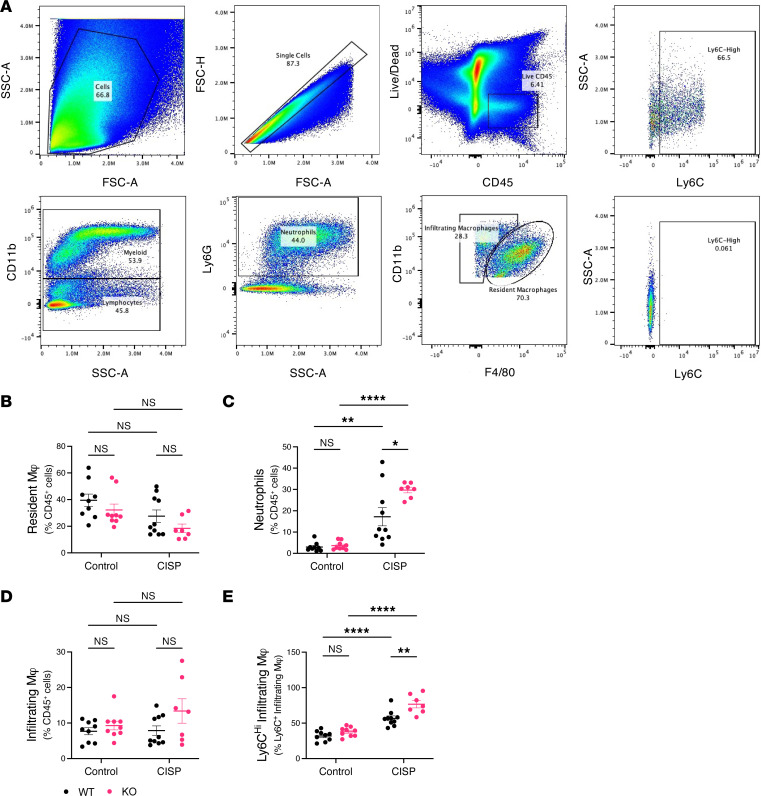
Enhanced immune response in VEGFR-3–specific RelA-deficient mice and floxed controls following inflammation-induced AKI. (**A**) Flow cytometry gating strategy for analyzing CD45^+^ leukocyte populations in kidneys from a cisplatin-treated knockout mouse (20 mg/kg), including neutrophils, kidney-resident macrophages (KRMs), infiltrating macrophages (IMs), and inflammatory Ly6c^hi^ IMs. (**B**) Quantification of KRMs (CD11b^int^, F4/80^hi^) as a percentage of total CD45^+^ cells in RelA-knockout and WT control mice. (**C**) Quantification of neutrophils (Ly6G^+^) as a percentage of total CD45^+^ cells. (**D**) Quantification of IMs (CD11b^hi^, F4/80^int^) as a percentage of total CD45^+^ cells. (**E**) Quantification of inflammatory Ly6c^hi^ IM cells as a percentage of total IMs (CD11b^hi^, F4/80^int^) (WT control, *N* = 9; KO control, *N* = 9; WT cisplatin, *N* = 10; KO cisplatin, *N* = 7). Cisplatin-treated mice with serum creatinine levels at least twice those of saline controls were included in this analysis. Graphs include data from 4 independent cohorts. *P < 0.0332, **P < 0.0021, ****P < 0.0001. WT, RelA^fl/fl^; KO, VEGFR-3^RelA–/–^.

To investigate immune response alterations in RelA-knockout mice compared with control floxed mice, we performed flow cytometric analysis, focusing on distinct innate immune cell populations: kidney-resident macrophages (KRMs) (F4/80^hi^, CD11b^int^), infiltrating macrophages (F4/80^int^, CD11b^hi^), neutrophils (LY6G^+^), and a subset of proinflammatory infiltrating macrophages (Ly6C^hi^, F4/80^int^, CD11b^hi^) (20, 21). The detailed gating strategy is outlined in Figure 3A.

Under baseline conditions (saline treatment), the percentage of KRMs within the CD45^+^ population was comparable between knockout and WT mice. However, following cisplatin-induced injury, the proportion of KRMs slightly decreased in knockout mice (Figure 3B), although this difference was not statistically different. This suggests that KRMs may have undergone cell death due to cisplatin injury and may be unable to self-renew and regenerate. There were no apparent differences between knockout and WT injured mice; this was expected as resident macrophages are present within the kidney prior to injury and do not require lymphatic vessels for tissue egress.

In contrast, knockout mice exhibited a robust increase in neutrophils at day 3 following cisplatin injury (Figure 3C). Moreover, while the total F4/80^int^, CD11b^hi^ infiltrating macrophage population (encompassing anti- and proinflammatory macrophage subpopulations) did not change significantly (Figure 3D), knockout mice exhibited a significant approximately 2-fold increase in Ly6C^hi^ proinflammatory macrophages (Figure 3E). These results suggested that a VEGFR-3 specific RelA knockout (a) resulted in greater susceptibility to injury and inflammation and/or (b) impaired lymphatic clearance of immune cells, preventing them from exiting the kidney through the collecting lymphatic vessels into the draining lymph nodes.

### Lymphatic transcriptional changes in RelA-knockout and floxed control mice.

**Figure 4:**
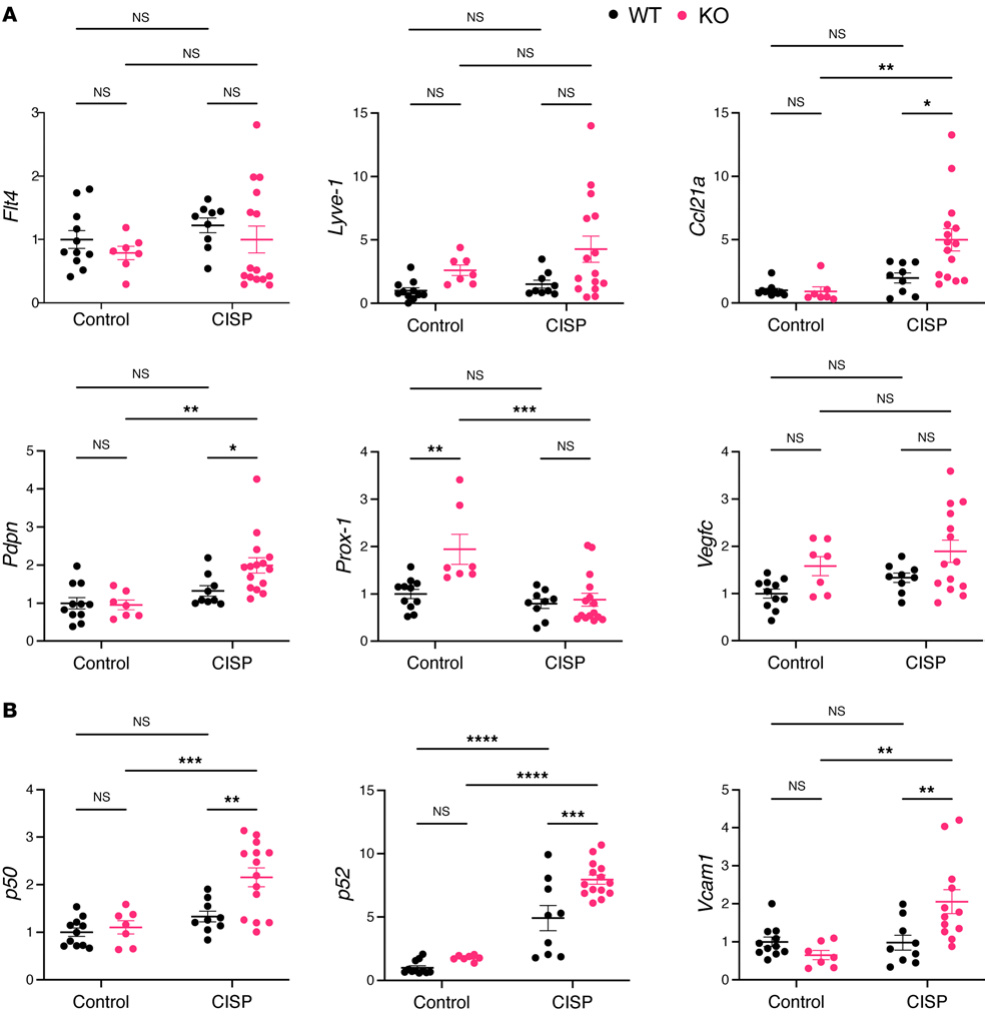
Transcriptional profiles of VEGFR-3–specific RelA-deficient mice and floxed controls following inflammation-induced AKI. (**A**) Lymphatic associated genes’ and (**B**) NF-κB and *Vcam1* genes’ expression analyses from kidney lysate, presented as fold change relative to tamoxifen+saline WT controls. Analysis included data from 4 independent knockout cohorts and 3 WT control cohorts (WT control, *N* = 11; KO control, *N* = 7; WT cisplatin, *N* = 9; KO cisplatin, *N* = 15 for *Flt4, Lyve-1, Ccl21a, Prox-1*, and *Pdpn*; *N* = 12 for *Vcam1*; *N* = 14 for *p50, Vegfc*, and *p52*). All transcript levels were normalized to *Gapdh*. Statistical significance (*P* < 0.05) was determined using 2-way ANOVA. *P < 0.0332, **P < 0.0021, ***P < 0.0002, ****P < 0.0001. WT, RelA^fl/fl^; KO, VEGFR-3^RelA–/–^.

We previously demonstrated that AKI models induce significant changes in lymphatic marker transcripts (22, 23). In the uninjured state, knockout mice showed no significant differences in *Flt4*, *Lyve-1*, *Ccl21a, Pdpn*, *Vegfc*, *p50*, and *p52* expression compared with WT controls (Figure 4). Most notably, cisplatin-treated knockout mice displayed significant upregulation (relative to controls) of canonical lymphatic markers (*Ccl21a* and *Pdpn*) compared with cisplatin-treated WT counterparts (Figure 4A). While these markers are not exclusively lymphatic endothelial cell-specific — with Pdpn being expressed by podocytes — these results strongly indicated accelerated transcriptional LA within 3 days of cisplatin injury in knockout mice, whereas WT mice did not. Although these LA markers were upregulated, *Prox-1* was significantly downregulated following cisplatin exposure in knockout mice. Notably, knockout mice exhibited greater *Prox-1* expression at baseline.

We further examined NF-κB subunits involved in canonical (*p50*) and noncanonical (*p52*) activation pathways alongside vascular cell adhesion molecule 1 (*Vcam1*). While we cannot attribute their expression specifically to lymphatics due to their ubiquitous presence in various kidney and immune cell types (particularly tubules), we can make conclusions about overall inflammation. Following injury, knockout mice exhibited significantly elevated levels of *p50, p52*, and *Vcam1*, indicating enhanced susceptibility to inflammation and possible compensation from the noncanonical NF-κB pathway (Figure 4B). The pronounced increase in *Vcam1* expression potentially signified defective repair processes in proximal tubules, suggesting compromised recovery mechanisms in knockout mice (24).

### Lymphatic protein alterations in RelA-knockout and floxed control mice.

**Figure 5:**
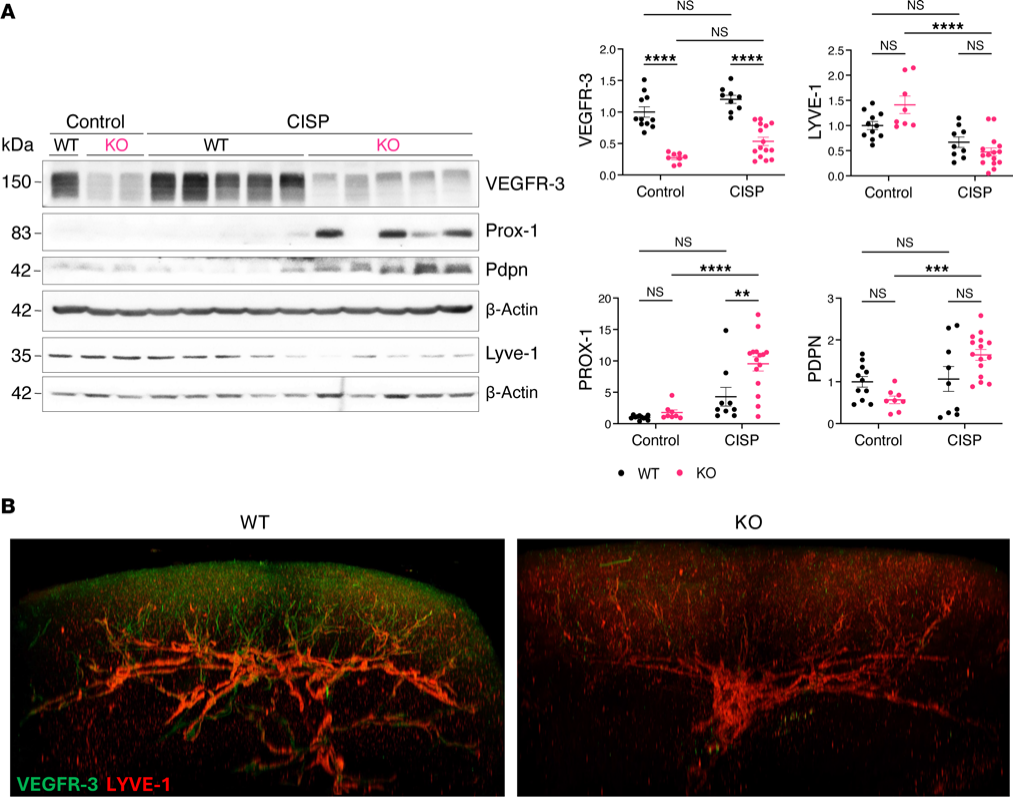
Lymphatic protein expression profiles in VEGFR-3–specific RelA-deficient mice and floxed controls following inflammation-induced AKI. (**A**) Analysis of lymphatic marker protein expression in kidney lysate by Western blotting. Protein expression levels were quantified using densitometry and normalized to either β-actin or GAPDH as loading controls. Representative blots are shown with corresponding quantification. Densitometric analysis includes data from 3 independent knockout cohorts and 3 WT cohorts (WT control, *N* = 11; KO control, *N* = 8; WT cisplatin, *N* = 9; KO cisplatin, *N* = 15). Statistical significance (*P* < 0.05) was determined using 2-way ANOVA. **P < 0.0021, ***P < 0.0002, ****P < 0.0001. (**B**) Visualization of lymphatic structures in cleared kidney tissue using confocal microscopy. Images show quarter-sectioned, optically cleared kidneys from both a WT control and knockout mouse. Lymphatic vessels are labeled with two markers: LYVE-1 (red) and VEGFR-3 (green). Images shown are maximum intensity projections that have been processed with denoising and deconvolution algorithms using NIS-Elements. Original magnification, ×10. WT, RelA^fl/fl^; KO, VEGFR-3^RelA–/–^.

To complement our transcriptional findings, we analyzed protein expression of key lymphatic markers using Western blotting. We observed a striking reduction in VEGFR-3 expression in knockout mice under both baseline and injury conditions (Figure 5A). This observation aligns with previous studies demonstrating direct regulation of VEGFR-3 expression by NF-κB in LECs (17). While both WT and knockout mice upregulated VEGFR-3 expression following cisplatin injury — consistent with expected inflammation-induced LA — the knockout mice started from a lower basal VEGFR-3 level and maintained significantly lower expression compared with controls after injury, indicating a blunted lymphangiogenic response.

Notably, LYVE-1 protein expression was significantly downregulated in cisplatin-treated knockout mice compared with baseline preinjury levels (Figure 5A). This reduction likely represents “real” decreased lymphatic expression rather than macrophage-derived signal, considering the substantial increase in infiltrating macrophages observed in knockout kidneys relative to controls (Figure 3). In contrast, expression of both PROX-1 and PDPN was significantly upregulated compared with saline baselines. Knockout injured mice exhibited higher PROX-1 levels compared with injured floxed counterparts (*P* < 0.05), suggesting a potential compensatory mechanism in response to LYVE-1 and VEGFR-3 downregulation (Figure 5A). These findings indicate that PROX1^+^ lymphatics may compensate to restore lymphatic architecture and promote differentiation of existing vessels.

### Immunolabeling of lymphatic markers in optically cleared kidney tissue.

To validate the Western blot results, we optically cleared and immunolabeled a quarter kidney with VEGFR-3 and LYVE-1 to definitively visualize lymphatic vessels in both knockout and control mice.

**Figure 6:**
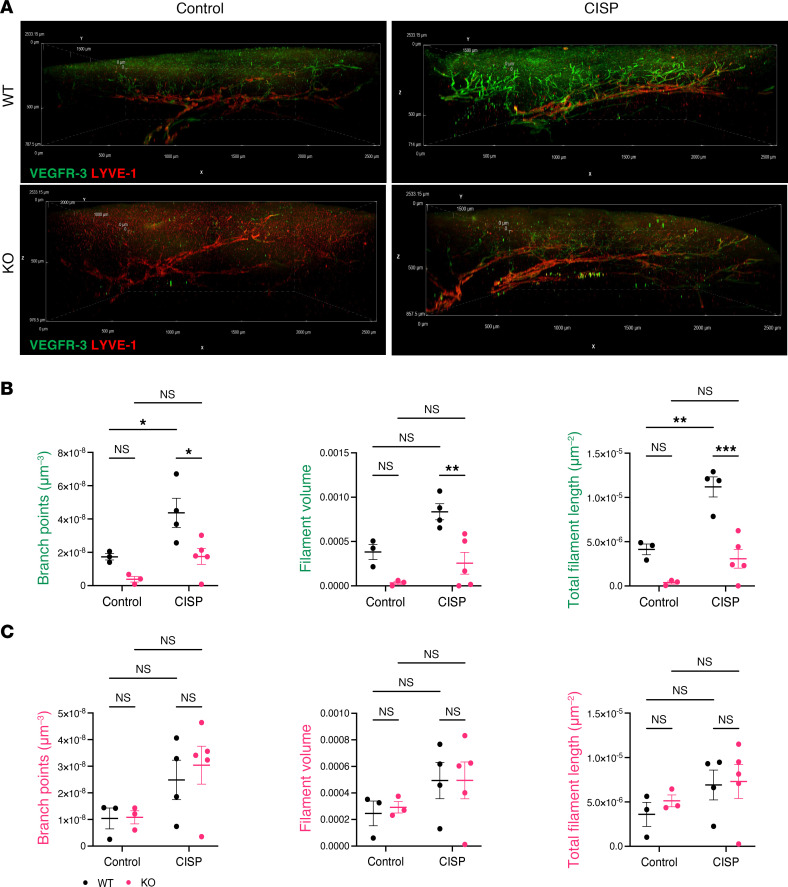
Visualization and quantification of lymphatic vessel features in VEGFR-3–specific RelA-deficient mice compared with floxed controls. (**A**) Representative confocal microscopy images (maximum intensity projections) of quarter kidney sections immunolabeled for VEGFR-3 (green, Alexa 647) and LYVE-1 (red, Alexa 594). Images compare VEGFR-3–specific RelA-deficient mice to floxed controls under baseline conditions and after cisplatin-induced AKI. 10× objective. (**B** and **C**) Quantitative analysis of lymphatic vessel morphology for (**B**) VEGFR-3^+^ and (**C**) LYVE-1^+^ vessels. Measured parameters include branch point density, total filament volume (μm^3^), and total filament length (μm). Analysis was performed using Imaris Filaments module following image processing (denoising, deconvolution, thresholding). All measurements were normalized to imaging volume based on image field dimensions: *x* axis (2,038 pixels), *y* axis (2,038 pixels), and *z* axis (section thickness in μm), with a pixel size of 1.24 μm/pixel (WT control, *N* = 3; KO control, *N* = 3; WT cisplatin, *N* = 4; KO cisplatin, *N* = 5). WT, RelA^fl/fl^; KO, VEGFR-3^RelA–/–^.

Injured knockout mice showed dramatically reduced VEGFR-3 expression compared with injured controls (Figure 5B) in both the hilar and cortical regions. Supplemental Videos 1 and 2 show 3D images of injured floxed and knockout mice, respectively. Comparative analysis between control and cisplatin-treated mice revealed striking differences in lymphatic architecture and marker expression across all experimental groups (Figure 6A and Supplemental Figure 4). Even under baseline conditions, knockout mice displayed substantially fewer VEGFR-3^+^ cortical branches, while WT mice maintained well-defined and continuous cortical lymphatic networks.

Following injury, WT mice demonstrated robust VEGFR-3–driven LA, with numerous small, developing branches predominantly present in the cortex (Figure 6A). In contrast, knockout mice showed minimal new branch formation and further reduced LYVE-1 expression.

An important observation was the prevalence of VEGFR-3^+^ (but LYVE-1^–^) cortical lymphatic branches in control mice (Supplemental Figure 5D). This supports the idea that LEC markers are more heterogeneously expressed throughout vessels than previously assumed. Our findings suggest that cisplatin-induced lymphatic expansion is primarily VEGFR-3 driven, while LYVE-1 expression is concentrated in collecting hilar lymphatic vessels.

### Quantification of immunolabeled cleared kidneys.

For quantitative analysis, we employed a comprehensive 3D analysis pipeline optimized by our laboratory. Binary masks were constructed from original maximal intensity projections for each channel, followed by segmentation using the filaments feature. We used an injured floxed mouse to demonstrate the complete analysis pipeline for both channels (Supplemental Figure 5, A–C).

Using Imaris software combined with machine learning algorithms, we quantified 5 key parameters from the binary masks of each channel: branch points, total lymphatic volume, total filament length, average mean diameter, and average segment length. The quantitative data confirmed our visual observations. Knockout control mice showed significantly reduced VEGFR-3^+^ lymphatic volume, branch points, and total filament length (Figure 6B). There were no statistically significant differences in VEGFR-3^+^ vessel mean diameter and segment length compared with those of WT controls (Supplemental Figure 6A). These findings corroborate that knockout mice have fewer VEGFR-3^+^ branch points and reduced total VEGFR-3^+^ volume and length, demonstrating that RelA plays a critical role in the formation, growth, and maintenance of VEGFR-3^+^ lymphatic vessels.

In contrast, LYVE-1 parameters showed markedly different patterns compared with VEGFR-3. LYVE-1 showed less robust changes, with no observed differences in branch points, filament volume, filament length, mean diameter, and segment length between floxed and knockout mice under injury conditions (Figure 6C and Supplemental Figure 6B). This suggests that RelA may also contribute to maintaining lymphatic vessel structural integrity in addition to promoting new VEGFR-3^+^ vessel growth.

## Discussion

Our study demonstrates the first targeted knockout of RelA expression specifically in VEGFR-3–expressing cells to our knowledge, enabling a precise characterization of its role during inflammation-induced AKI. While previous research by Flister et al. (15) explored the effects of NF-κB1 (p50), their global knockout model had limited conclusions about the specific role of NF-κB in the lymphatic system. Our work reveals that deletion of RelA in VEGFR-3^+^ cells exacerbates kidney function deterioration and histopathological disruption following inflammation-induced AKI. It also significantly modulates VEGFR-3–mediated LA and expression and augments the acute inflammatory response.

Our results contrast with those of a previous study showing that pharmacologic inhibition of NF-κB using pyrrolidine dithio-carbamate ammonium (PDTC) ameliorated kidney function following folic acid–induced AKI, as evidenced by improved serum creatinine levels. This discrepancy likely stems from PDTC’s nonselective nature, which inhibits IκB and consequently affects both p50 and p65 subunits across multiple cell types. The benefits observed in that study may also be attributed to effects unrelated to lymphatics. Complementary studies have established that NF-κB and its precursors play crucial roles in maintaining kidney function and regulating renal fibrosis (25, 26). However, contradictory evidence demonstrates the dual nature of NF-κB — both partial inactivation and activation have been linked to hypertension and CKD (26). Therefore, optimal regulation of NF-κB is critical for kidney health. It is logical that overactivation of a master transcriptional regulator like NF-κB would increase immune cell recruitment, activate inflammatory pathways, and enhance reactive oxygen species production. These downstream effects ultimately mediate organ injury through various cell death mechanisms (26). Therefore, targeting NF-κB signaling pathways offers promising therapeutic potential for fibrosis and inflammation-driven kidney diseases.

VEGFR-3 is essential for lymphatic development and LA. Liu et al. investigated the role of VEGFR-3 in kidney lymphatics during embryogenesis and postnatal development by mutating its kinase activity. During development, mice with heterozygous or homozygous VEGFR-3 mutations displayed reduced kidney lymphatics or complete absence of lymphatics, respectively. Interestingly, while heterozygous mice showed reduced lymphatic vessel density, they maintained normal kidney function both before and after cisplatin-induced injury, though histology showed increased perivascular inflammation (27). The apparent contradiction between Liu’s findings and ours likely stems from methodological differences. They altered VEGFR-3 kinase activity via a missense mutation, whereas our work fully deleted RelA in VEGFR-3–expressing cells. The robust decrease in VEGFR-3 expression in our model may relate more to changes in total protein expression rather than kinase activity. Furthermore, use of RelA to drive the deletion of VEGFR-3 may also be an important distinction, particularly in the context of the response of LECs to inflammation, and responses other than VEGFR-3 deletion that have yet been identified. This discrepancy highlights that RelA, in addition to VEGFR-3, likely plays important roles beyond lymphatic development, potentially affecting inflammatory responses critical during acute injury.

VEGFR-3 activation by ligation with VEGF-C or VEGF-D is necessary for initiating LA. NF-κB and PROX-1, a key transcriptional regulator of LA, work synergistically to activate VEGFR-3 in hdLECs (17). Our results provide strong evidence that VEGFR-3–mediated LA and VEGFR-3 expression were nearly abolished in the knockout model. We observed minimal cortical lymphatic expansion positive for VEGFR-3, suggesting that RelA is essential for the expression of this lymphangiogenic marker.

Prox-1 exhibited differing expression patterns at the gene and protein levels. In injured knockout mice, *Prox-1* RNA expression was downregulated while PROX-1 protein expression was upregulated, suggesting that its translation is critical for initiating the lymphangiogenic cascade (22, 28, 29). These distinct expression profiles may reflect the inherent temporal disconnect between RNA and protein expression patterns — at day 3 after injury, mRNA is downregulated while protein expression is upregulated. Unlike many lymphatic system cell-associated genes, Prox-1 expression may be regulated primarily through posttranscriptional mechanisms, including epigenetic modifications (30, 31). Although both *Pdpn* and *Ccl21a* are transcriptionally upregulated following injury, our data suggest these genes may not be fully under the control of *Prox-1*, the master lymphatic transcriptional regulator. It is possible that only *Flt4* and *Lyve-1* are under the direct regulation of *Prox-1*.

While total protein for both markers, VEGFR-3 and LYVE-1, was downregulated following inflammation-induced injury, PDPN and PROX-1 protein expression increased, albeit insufficiently to restore kidney function, suggesting a compensatory lymphangiogenic response driven by alternative pathways independent of VEGFR-3. This differential marker expression likely reflects the inherent heterogeneity of LECs (32) within the kidney, where distinct LEC subpopulations or subtypes may exhibit varying dependence on RelA signaling pathways and respond differently to inflammatory stimuli. This could explain the selective upregulation of PROX-1 and PDPN lymphatic markers despite VEGFR-3 suppression. While there appeared to be a compensatory effect, it was insufficient to preserve kidney function comparable to control mice, indicating that RelA-dependent VEGFR-3–driven LA is critical for protection against kidney injury. This finding aligns with those in our previous studies demonstrating that inhibiting VEGFR-3 expression via the MAZ51 pharmacologic inhibitor exacerbated kidney dysfunction following cisplatin injury (8).

Interestingly, our in vitro data in hdLECs revealed that silencing p65 and p50 subunits upregulated lymphatic marker RNA expression, including *Flt4,* which has been recently shown to be differentially expressed in kidney and skin (33). This contrasts with our in vivo findings where VEGFR-3 expression decreased in RelA-knockout mice both before and after injury. This apparent discrepancy likely reflects several key differences between in vitro and in vivo model systems: (a) species and organ-specific differences between *human dermal* LECs and *murine kidney* LECs; (b) the in vitro use of PDPN^+^ and CD31^+^ sorted hdLECs, which inherently enriches for a specific LEC subset that may exclude VEGFR-3^+^ populations and fails to capture the heterogeneous LEC populations present in kidney tissue; and (c) the absence of complex inflammatory microenvironments, cell-cell interactions, and paracrine signaling in vitro that are critical drivers of in vivo injury responses.

Our 3D imaging analysis and quantification revealed striking heterogeneity between VEGFR-3 and LYVE-1^+^ lymphatic vessels, which challenges the traditional belief that these LEC markers are homogeneously expressed throughout lymphatic networks. The differential responses — with VEGFR-3 showing dramatic reduction while LYVE-1 exhibited comparable increases in branch points and volume in injured knockout mice compared with WT mice (Supplemental Figure 6B) — suggest these markers define functionally distinct lymphatic vessel populations within the kidney. Importantly, our image analysis protocol required manual adjustment of absolute intensity thresholds for each mouse using the surfaces tool to generate accurate binary masks. This critical step ensured appropriate isolation of lymphatic vasculature while excluding background signal. Therefore, our quantitative results represent the morphological characteristics of detectable vessels following threshold optimization, rather than total protein expression levels.

Detailed segmentation and skeletonization analysis of LYVE-1 and VEGFR-3 structures revealed differences in both branching architecture and spatial distribution patterns, further supporting the heterogeneous nature of lymphatic marker expression (Supplemental Figure 5D). In knockout mice, we observed substantially fewer VEGFR-3^+^ branch points and cortical lymphatic vessels compared with those in floxed mice. Following inflammation-induced AKI, knockout mice exhibited disrupted lymphatic networks, with the remaining LYVE-1– and VEGFR-3–expressing vessels showing irregular, fragmented patterns predominantly confined to cortical regions. In contrast, WT mice maintained organized and continuous lymphatic networks throughout the kidney, with particularly robust and well-developed VEGFR-3^+^ vessel networks that extended from the hilum into the cortical regions.

The apparent discrepancy between Western blot data and 3D imaging quantification for the LYVE-1 signal may be attributed to several factors. First, immunofluorescence captures intact LYVE-1^+^ lymphatic vessels without regard to level of LYVE-1 expression and in relation to tissue volume, while Western blotting measures total protein content, including degraded and intracellular proteins. Second, these findings suggest an adaptive mechanism where LYVE-1 protein is distributed across a larger vessel area. Specifically, knockout mice might have altered lymphatic vessel architecture that compensates for surface area despite having reduced total protein. The observed increase in filament volume and number of detected points in knockout mice relative to their control counterparts serves as a proxy measurement for increased branching and de novo LA.

Both visual assessment and quantitative analysis confirmed that lymphatic highways were disrupted in knockout mice. One primary function of the lymphatic system is facilitating immune cell trafficking, including macrophages and dendritic cells, from interstitial tissues to draining lymph nodes and, ultimately, back to the central venous system. When these lymphatic highways become “leaky” or discontinuous, they no longer permit immune cell egress, leading to accumulation of macrophages and dendritic cells in inflamed tissues. This accumulation perpetuates inflammatory signaling and exacerbates tissue damage. We propose that the impaired LA and downregulation of VEGFR-3 in our model prevent infiltrating macrophages from exiting the kidney after injury. This hypothesis is supported by the significant increase in Ly6c^hi^ infiltrating macrophages and neutrophils observed in knockout kidneys following cisplatin injury. This pattern suggests impaired immune cell egress from injured kidneys in the knockout model. Importantly, KRMs remained relatively unchanged across both groups, which is expected, as these cells constitute the majority of CD45^+^ cells (20, 34–37) and are present in the kidney prior to injury. The preservation of resident macrophages in both groups supports our hypothesis that the observed changes are more likely to reflect impaired trafficking rather than generalized immune dysfunction. These observations might also indicate an overall increased susceptibility to injury in knockout mice.

While our study reports significant findings regarding the role of NF-κB in LA, several limitations remain. An important consideration that is often overlooked is the temporal dynamics of RelA regulation throughout the injury-repair timeline. While our work investigates the role of RelA during the early injury phase (day 3 after AKI), the contribution of canonical NF-κB signaling may differ during later stages of injury and recovery. In this work, we investigated only an AKI model; future studies should investigate the temporal dynamics of LA and use other models, like bilateral ischemia/reperfusion and chronic kidney models, as we have done in the past (22). These experimental models would capture the AKI-to-CKD transition, which would be valuable for defining the optimal therapeutic window for targeting canonical NF-κB signaling.

In addition, our model only targets RelA deletion in VEGFR-3–expressing cells. While VEGFR-3 is predominantly expressed by LECs, it is also expressed by ascending vasa recta, glomerular endothelial cells, and macrophages, meaning the model is not entirely LEC specific (38–40). Different subunits of NF-κB and VEGFR-3 have established roles in macrophage biology (40–42). Deletion or dysregulation of these key genes can impair macrophage function (e.g., phagocytosis and innate immune defense), polarization, and cell-cell interactions. The phenotype we observe — accumulation and impaired egress of proinflammatory Ly6C^hi^ macrophages in knockout mice — may result from decreased VEGFR-3 expression in macrophages themselves. This introduces a layer of complexity, as it remains incompletely understood whether our findings reflect disruption of lymphatic drainage pathways or functional and cellular changes in macrophages that impair their ability to exit the kidney. Similarly, we acknowledge that there is no direct mechanistic evidence showing that RelA transcriptionally regulates VEGFR-3; instead, our work focused on the functional role of NF-κB signaling in lymphatic responses following injury. Beyond its role in macrophage biology, NF-κB dysregulation and its knockout may also affect mitochondrial function in VEGFR-3–expressing cells. This is particularly relevant given that LA depends on fatty acid oxidation (43).

Additionally, in our model, RelA knockout is not limited to kidney tissue. Systemic lymphatic dysfunction could indirectly influence kidney function outcomes that are independent of renal lymphatics. Finally, while we allude to potential changes in lymphatic function in our knockout model, we cannot make conclusive statements about function. Further studies tracking and labeling immune cells can help elucidate if lymphatic transport is affected. Recent studies have highlighted organ-specific features of renal lymphatic vessels (33). Importantly, anatomy-based radiologic imaging can identify pathologies involving lymphatic vessels that may improve diagnosis and treatment strategies for these conditions (44).

This study provides potentially novel insights into the molecular regulation of kidney LA and underscores the importance of lymphatic endothelial cell-specific NF-κB signaling in kidney injury responses. Further investigation of these pathways may lead to innovative therapeutic approaches for AKI and potentially other inflammatory kidney diseases.

## Methods

### Sex as a biological variable.

Only male mice were included in this study because they exhibit greater susceptibility to AKI and provide a more robust model for investigating injury-induced lymphatic responses.

### hdLECs in hypoxic conditions.

hdLECs (PromoCell, C-12217) were cultured in Endothelial Cell Basal Medium MV2 (PromoCell, C-22221) supplemented with essential growth factors (PromoCell, C-39221) in T75 flasks. Cells were seeded in 6-well plates and were grown to 80% confluency. The plates were then placed in hypoxia chambers and exposed to 1% O_2_ for approximately 3 minutes before being moved to the incubator and stored at 37°C in hypoxic conditions for various time points. At each time point, the hypoxia chambers were opened and cells were washed with PBS and then collected using a cell scraper. The collected cells were immediately lysed with 500 μL of TRIzol for RNA extraction.

### Animals.

Nine to 13-week-old male Flt4^Cre^xRelA^flox^ (in-house) and RelA^fl/fl^ mice (Jackson Laboratory; strain 024342) on a C57BL/6J background were used for this study. The Flt4^Cre^xRelA^flox^ strain was generated by crossing Vegfr3-CreERT2 mice (45) with RelA^fl/fl^ mice, resulting in VEGFR-3^RelA–/–^ mice (Figure 1A) (46). RelA^fl/fl^ mice (WT) served as experimental controls.

Tamoxifen (0.15 mg/g body weight dissolved in corn oil) was administered intraperitoneally for 3 consecutive days to induce Cre-mediated recombination. Successful recombination resulted in the excision of RelA exon 1 and activation of GFP expression (46). Following a 10- to 11-day washout period, mice received a single intraperitoneal injection of either cisplatin (20 mg/kg) or saline (control). Three days later, mice were anesthetized with isoflurane, exsanguinated, and perfused with filtered, ice-cold PBS followed by 4% paraformaldehyde (PFA). To optimize tissue preservation for different analytical methods, 1 kidney was collected immediately following PBS perfusion to preserve protein structure and RNA integrity, while the contralateral kidney subsequently underwent PFA perfusion for histological and immunofluorescent imaging. Kidney and liver tissues were harvested and flash-frozen for downstream analyses.

### Lymph node isolation and characterization.

Mice were anesthetized with ketamine/xylazine based on body weight. 25 microliters of 5% Evan’s blue dye (Sigma) were dissolved in 0.9% Sodium Chloride (Hospira Inc.), and the mixture was injected into the rear footpad using a syringe with a 26.5 g needle (BD) pointed in the caudal direction. 10 minutes were allowed for the dye to diffuse systemically and permeate through the lymphatics. The renal lymph node and collecting lymphatic vessel attached to the lumbar lymph node were visualized, resected, and digested with TRIzol. The livers were also collected to confirm knockout via *eGFP* RNA expression.

### Kidney function measurements.

To assess kidney function, serum creatinine levels and GFR were measured on day 3 following injury. Blood was collected via facial vein bleed or terminal cardiac puncture. Serum creatinine was also analyzed immediately after the 3 consecutive doses of tamoxifen and collected via facial vein bleed to ensure it did not affect kidney function. Serum creatinine was measured using liquid chromatography–mass spectrometry at the University of Alabama at Birmingham –UCSD (UAB-UCSD) O’Brien Center for AKI Research bioanalytical core facility.

To determine GFR, mice were lightly anesthetized with 1.5% isoflurane, and a transdermal monitor (MediBeacon) was placed on the shaved dorsal flank of the mouse. Mice were injected via the tail vein with 100 μL FITC-sinistrin (25 mg/mL) dissolved in saline. Monitors were removed after 2 hours and clearance of FITC-sinistrin was assessed using the MediBeacon software (22).

### Histology.

Mice were anesthetized and perfused with 10 mL cold, filtered PBS followed by 10 mL cold, filtered 4% PFA. The right kidney was harvested, decapsulated, and sectioned transversely in the center. The middle section was fixed in 10% neutral buffered formalin for 24 hours at room temperature, transferred to 70% ethanol, and stored at 4°C until paraffin embedding. Sections (5 μm) were then stained with periodic acid–Schiff (PAS). The two remaining kidney poles were fixed in 4% PFA/PBS to later be used for tissue clearing.

PAS-stained kidneys were imaged using the Keyence BZ-X710 microscope (Keyence Corporation) at ×20 magnification and scored in a blinded fashion by a nephrologist. 10 random fields within the whole kidney were imaged, and the scores from each field were averaged. Kidney damage was assessed for tubular casts, tubular necrosis, and brush border loss on a scale of 0–4: 0 (no damage), 1 (≤25% area), 2 (25%–50% area), 3 (50%–75% area), and 4 (>75% area) (22).

### Real-time quantitative PCR.

The left kidney was collected after perfusion with 10 mL PBS and decapsulated. The kidney was bisected, with one-half designated for RNA analysis and the other for protein studies, and then immediately flash-frozen. A section of the right liver lobe was also harvested and flash-frozen for subsequent *eGFP* RNA expression analysis. Both kidney and liver tissues were processed by sonication and lysed in 1 mL TRIzol (Invitrogen, 15596026).

RNA from all samples was extracted and reverse transcribed to cDNA using the QuantiTech kit (Qiagen, 205311). Quantitative PCR was performed using PowerUP SYBR Green Master Mix (Thermo Fisher, A25741) on a StepOnePlus Real-Time PCR System (Thermo Fisher, 4376357). All primer sequences are listed in Supplemental Table 1.

### Western blotting.

One-half of the PBS-perfused kidney was homogenized in RIPA buffer (10 mM Tris-HCl, 5 mM EDTA, 150 mM NaCl, 10% NP-40, 10% Triton X-100) containing protease and phosphatase inhibitors (100× dilution) (8). After sonication, protein concentration was determined by a bicinchoninic acid assay. Samples (75 μg/lane) were separated by SDS-PAGE and probed with the following antibodies: VEGFR3/Flt-4 (1:1,000; R&D Systems, AF743), mouse Podoplanin (1:5,000; R&D Systems, AF3244), Prox1 (1:1,000; Abcam, ab199359), Lyve-1 (1:800; Novus Biologicals, NB600-1008), phospho-NFκB p65 (1:1,000; Cell Signaling, 3033), total NF-κB p65 (1:2,500; Cell Signaling, 8242), HIF-1α (1:1,000; Cayman, 10006421), β-actin (1:10,000; Sigma, A2228), and GAPDH (1:10,000; Millipore, MAB374). Densitometry was performed using Image Studio Lite version 5.2.

### Immunolabeling for 3D visualization of lymphatics in cleared kidney tissue.

After perfusion with filtered PBS and 4% PFA, kidneys were harvested, decapsulated, cut into halves, submerged in cold 4% PFA, and incubated at 4°C for 72 hours. 4% PFA was then changed to 0.25% and stored long-term at 4°C. We developed an antibody-based 3D lymphatic imaging strategy for adult mouse kidneys by optimizing the iDISCO protocol (47, 48). Kidneys designated for tissue clearing were sectioned both transversally and sagittally to obtain one-quarter sections.

After a series of methanol dehydration/rehydration, delipidation with organic solvents (e.g., dichloromethane), permeabilization, blocking, and washing, kidneys were immunolabeled with LYVE-1 (1:200; Novus Biologicals, NB-600-1008) and VEGFR-3 (1:200; R&D Systems, AF743) for 96 hours at 37°C. Tissues were subsequently washed and incubated with secondary antibodies (1:300 dilution; AF594 and AF647 for LYVE-1 [Novabus, nb-6008] and VEGFR-3 [R&D Systems, AF743], respectively) for 96 hours at 37°C protected from the light. After additional washes with Ptwh (PBS containing 0.2% Tween-20 and 10 mg/L heparin), kidneys were again dehydrated, delipidated, and incubated in Dibenzyl Ether (Thermo Scientific Chemicals, AC148400010; refractive index = 1.424). Kidneys were imaged using confocal microscopy promptly following immunolabeling and clearing.

### Confocal microscopy.

Images with a pixel size of 1.24 (μm/px) were captured with a Nikon A1R Confocal microscope with the Nikon air ×10 objective (Plan Apo λ ×10, wd 4000, refractive index 1.0, NA 0.45) at the UAB High Resolution Imaging Facility. A sequential resonant scanner was used. The emission and excitation wavelengths for AF594 (LYVE-1) and AF647 (VEGFR-3) were set to 595/561.7 nm and 700/637.9 nm, respectively. Tissue images were captured by stitching 2 × 2 tiles with a 1% overlap using the Nikon NIS-Elements software. First, a transverse cut was made to section the kidney in half, followed by a longitudinal cut through the one half. The images represent one-fourth of a full tissue section. Postprocessing included noise reduction with Denoise AI and image deconvolution using the Nikon NIS-Elements software. Images in side-by-side comparisons in Figure 5B, Figure 6A, and Supplemental Figure 3 were adjusted using identical LUT settings to ensure accurate fluorescence intensity comparisons.

### Image analysis and lymphatic vessel quantification.

All computational analyses were performed using Imaris (10.2.0) software. Segmentation was applied to define boundaries and create binary masks for each channel (AF594 and AF647). Before implementing segmentation and machine learning techniques, thresholds for each animal’s lymphatic vasculature were manually calibrated. The “Filament Tracing” 3D segmentation module quantified total lymphatic volume, mean segment diameter, and number of branch points. Microvascular structural measurements were calculated using the “Filament Tracing” module. For lymphatic vessel analysis, denoised/deconvolved files were converted to Imaris file format (48).

Briefly,.ims files were imported into Imaris, and LUTs were optimized for each mouse. A surface module was created for both VEGFR-3 and LYVE-1 channels. Absolute intensity thresholds were adjusted individually for each mouse to account for background variation. Standardized parameters (“Number of voxels Img=1” and “Sphericity”) were applied across all mice. Finally, background elements that were nonlymphatic were manually deleted using the pencil tool. From this surface, a binary mask was generated. Next, a filament module was created for each channel. Diameter parameters were defined as 1 μm (minimum) and 30 μm (maximum). The detection type settings were set to exclude the soma and spines of filaments (48). Once the filament algorithm was trained, the settings were applied to all other mice uniformly. All parameters were optimized using an injured mouse. Comprehensive panels of statistics were exported directly from the software and analyzed in Prism 9.4.0.

### Analytical flow cytometry.

CD45^+^ immune cells were isolated as previously described (20, 21, 36, 49). Mice were anesthetized and perfused with 10 mL cold PBS, and kidneys were harvested and decapsulated. Kidneys were finely minced and digested in collagenase B (1 mg/mL in RPMI) for 30 minutes at 37°C. The digestion reaction was terminated with cold PEB (PBS, 2 mM EDTA, 1% wt/vol BSA). The tissue was disaggregated, passed through an 18-gauge syringe, and filtered through a 40 μm strainer. After red blood cells were lysed and tissue aggregates were washed with PBS, cells were stained with violet fixable viability dye (Invitrogen, L34955) and anti-CD16/32 antibody and then subsequently stained using anti-CD45.2 BV650 (BioLegend, 109836), anti-F4/80 APC-ef780 (Invitrogen, 47-4801-82), anti-CD11b SB600 (Invitrogen, 63-0112-82), anti-CD8 PerCP (BD Biosciences, 3896), anti-CD161 PE (eBioscience, 12-5941-83), anti-Ly6G AF700 (BioLegend, 127622), anti-CD4 FITC (Invitrogen, 11-0041-82), anti-TCRb PE-Cy5 (BD Biosciences, 553173), anti-Ly6C APC (eBioscience, 17-5932-82), CD106 PerCP-Cy5.5 (BioLegend, 105715), and CD19 SB 702 (Invitrogen, 67-0193-82). Results were analyzed using FlowJo (v.10.0.0).

### Statistics.

GraphPad Prism 9.4 was used for statistical analysis. The mean ± SEM was plotted, and unpaired 2-tailed *t* tests, 1-way ANOVA, or 2-way ANOVA was used to determine the statistical significance between experimental groups. Šídák’s and Tukey’s HSD post hoc analyses were conducted following 1-way and 2-way ANOVA tests, respectively. An α value of 0.05 was used to determine significance.

### Study approval.

All animal procedures were performed in accordance with NIH guidelines regarding the care and use of live animals and approved by the Institutional Animal Care and Use Committee of the UAB (APN 21073, approved from 2023 to 2026).

### Data availability.

Numerical values from all graphs can be found in the Supporting Data Values file.

## Author contributions

ALM, AMT, GGR, and AA conceptualized and designed the study. ALM drafted this manuscript. ALM, AAZ, KHM, AMT, GGR, YJ, SE, and HJ performed experiments. ALM, AMT, KHM, AAZ, GGR, TCL, JFG, and AA interpreted the results of the experiments. ALM, AMT, AAZ, KHM, GGR, BJM, TCL, JFG, and AA performed review and revision of the paper. All authors read and approved the final paper.

## Funding support

This work is the result of NIH funding, in whole or in part, and is subject to the NIH Public Access Policy. Through acceptance of this federal funding, the NIH has been given a right to make the work publicly available in PubMed Central.

- NIH grants U2C DK133422 and TL1 DK139566 (to ALM), T32-GM008361-30 (to UAB Medical Scientist Training Program and ALM), U54 DK137307 (to UAB-UCSD O’Brien Center for Acute Kidney Injury Research and AA), and 1F30DK135349-01A1 (to GGR).
- Department of Veterans Affairs grant 5I01BX004047-34003797 (to TCL).

## Supplementary Material

Supplemental data

Unedited blot and gel images

Supplemental video 1

Supplemental video 2

Supporting data values

## Version 1 01/23/2026

In-Press Preview

## Version 2 03/09/2026

Electronic publication
