# Supplementary material for: NF-κB–driven lymphangiogenesis affects kidney function via a VEGFR-3–mediated pathway
Source: JCI Insight. 2026 Jan 22;11(5):e198992. doi: 10.1172/jci.insight.198992 (PMC13041690; doi:10.1172/jci.insight.198992)
Supplement: Supplemental data [file jciinsight-11-198992-s191.pdf]

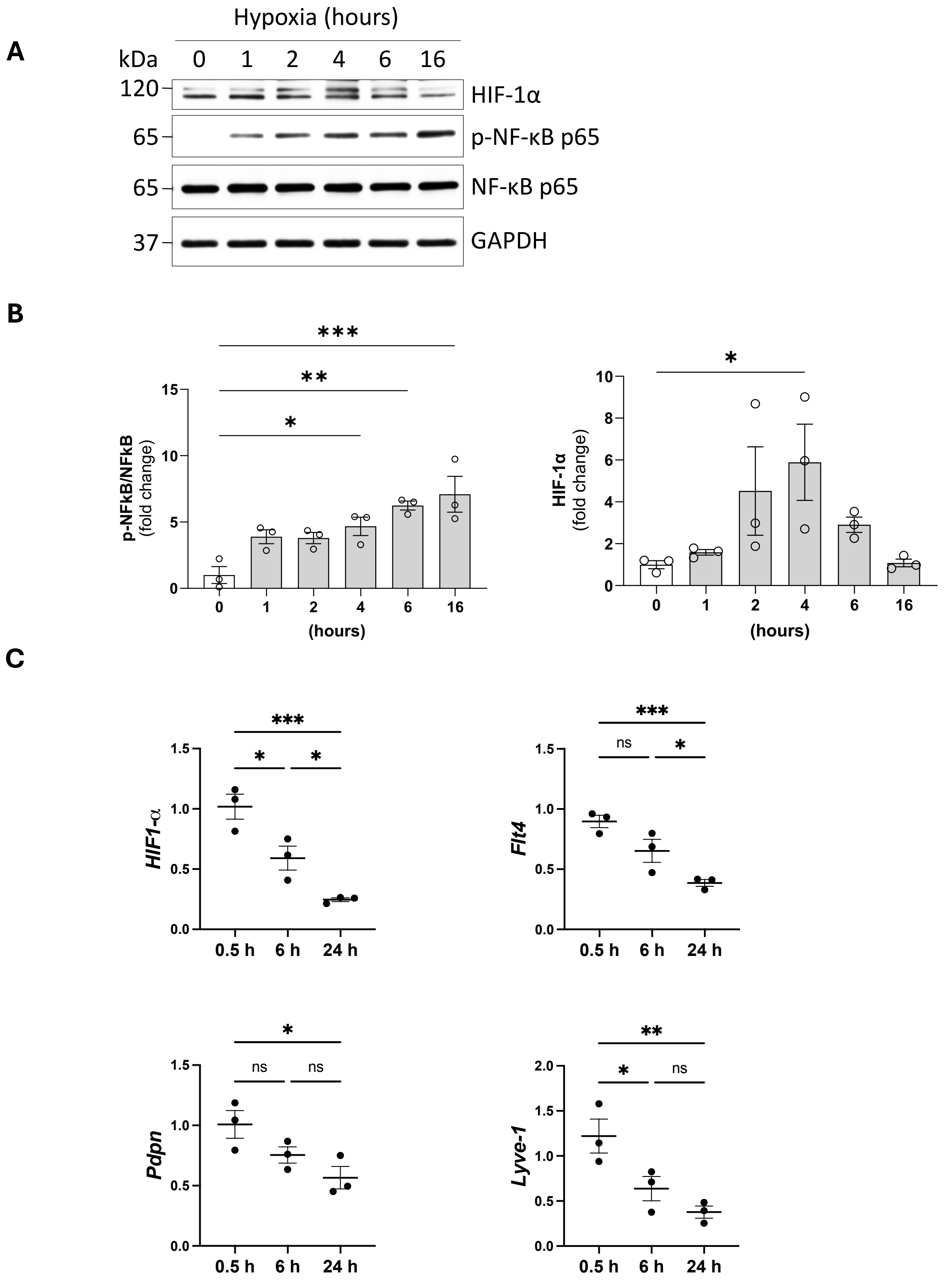
A
Hypoxia (hours)
kDa 0 1 2 4 6 16
120
HIF-1α
65
p-NF-κB p65
65
NF-κB p65
37
GAPDH
B
p-NFkB/NFkB (fold change)
(hours)
HIF-1α (fold change)
(hours)
C
HIF1-α
Flt4
Pdpn
Lyve-1
0.5 h
6 h
24 h
ns

Supplemental Figure 1: Effects of Hypoxia-Induced Inflammation and NF-κB Knockdown on Lymphatic Marker Expression in Human Dermal Lymphatic Endothelial Cells (hdLECs).

(A) Western blot analysis was performed to assess the success of hypoxic conditions at 0, 1, 2, 4, 6, and 16 hours by probing for hypoxia-inducible factor-1alpha (HIF-1α), phosphorylated p65, and total levels of p65. (B) Densitometric analysis of protein expression was conducted, comparing phosphorylated p65 (p-p65) to total p65 and HIF-1α normalized to GAPDH. 2-way ANOVA tests were used to determine significance ($p < 0.05$). (C) RNA expression of *HIF-1α* and lymphatic markers was measured at 0, 0.5, 6, and 24 hours in hdLECs exposed to 1% O_2 hypoxia. Expression levels were normalized to *Gapdh* and normoxic controls. 1-way ANOVA tests were used to determine significance ($p < 0.05$).

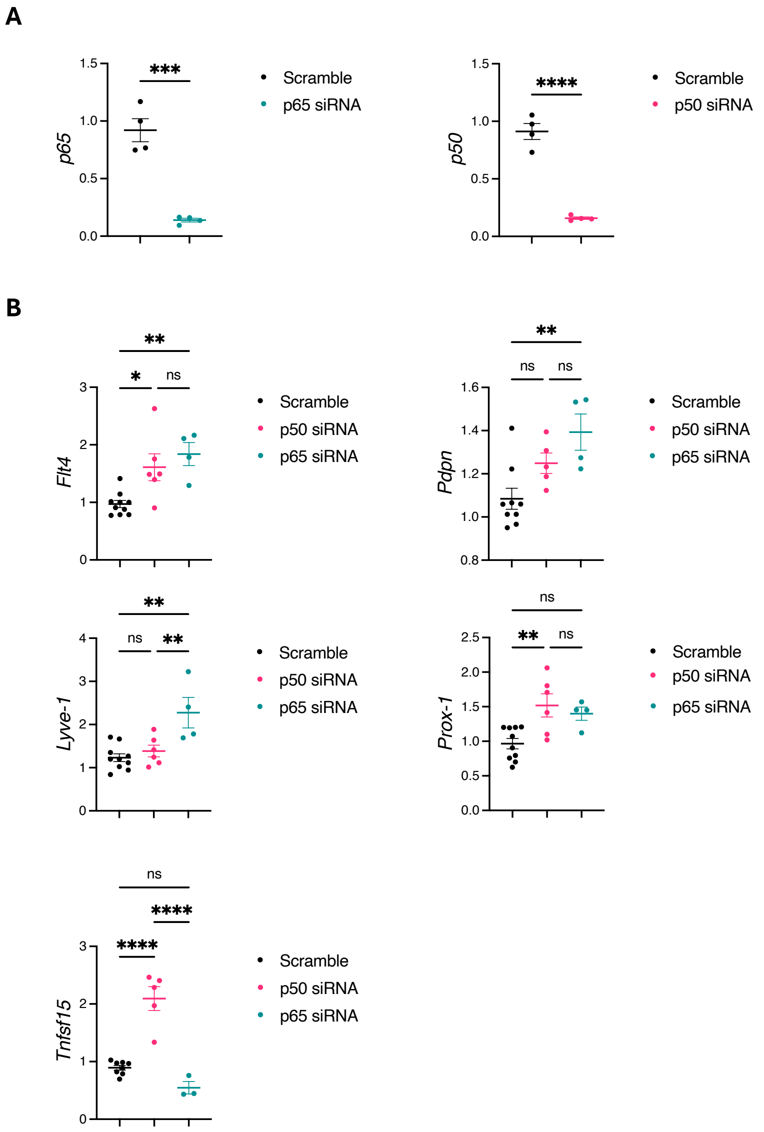

Supplemental Figure 2: Knockdown of NF-κB1(p50) and RelA (p65) subunits of NF-κB in human dermal lymphatic endothelial cells (hdLECs).

(A) Validation of p50 and p65 siRNA knockdown efficiency by RT-qPCR, with expression levels normalized to *Gapdh* and untransfected controls. Values represent fold change relative to untransfected controls. Unpaired t-tests were used to determine significance ($p < 0.05$). (B)

RNA expression of lymphatic-associated markers in hdLECs transfected with p50, p65, or scramble siRNA normalized to *Gapdh* and untransfected controls. 1-way ANOVA tests were used to determine significance ($p < 0.05$).

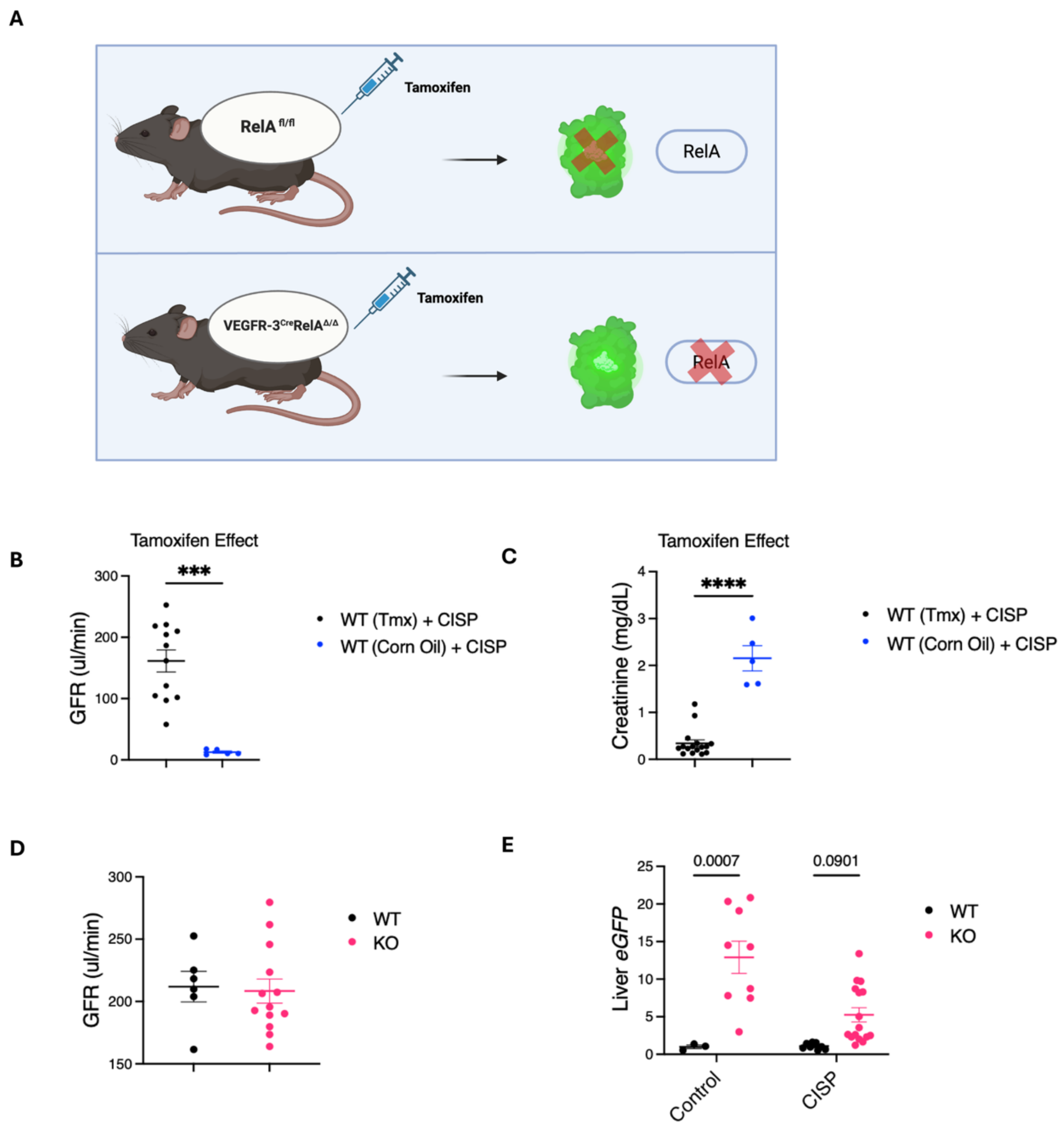

Supplemental Figure 3: Tamoxifen protects against acute kidney injury.

(A) Schematic illustrating the predicted biological response following tamoxifen administration and activation of the Cre-loxP system. (B) WT mice that received three intraperitoneal injections of tamoxifen (0.15 mg/g) (N=12) had higher glomerular filtration rates and were more protected from developing AKI compared to mice that received vehicle (corn oil) (N=5). (C) WT mice that received three intraperitoneal injections of tamoxifen (0.15 mg/g) (N=16) had lower serum

creatinine measurements and were more protected from developing AKI compared to mice that received vehicle (corn oil) (N=5). (D) Baseline glomerular filtration rates were measured to show that transgenic mice (VEGFR-3^{Cre}) (N=13) had comparable baseline kidney function compared to WT (VEGFR-3 negative Cre) (N=6) mice prior to injury. Statistical significance ($p < 0.05$) was determined by unpaired t-test analysis. (E) Hepatic *eGFP* RNA expression analysis relative to WT mice and normalized to *Gapdh* confirming successful gene knockout in tamoxifen-treated mice compared to WT controls. Sample sizes (WT control, KO control, WT cisplatin, KO cisplatin): N=3, 9, 9, 16. P-values were determined with a 2-way ANOVA test. WT= $RelA^{fl/fl}$, KO= VEGFR-$3^{RelA-/-}$.

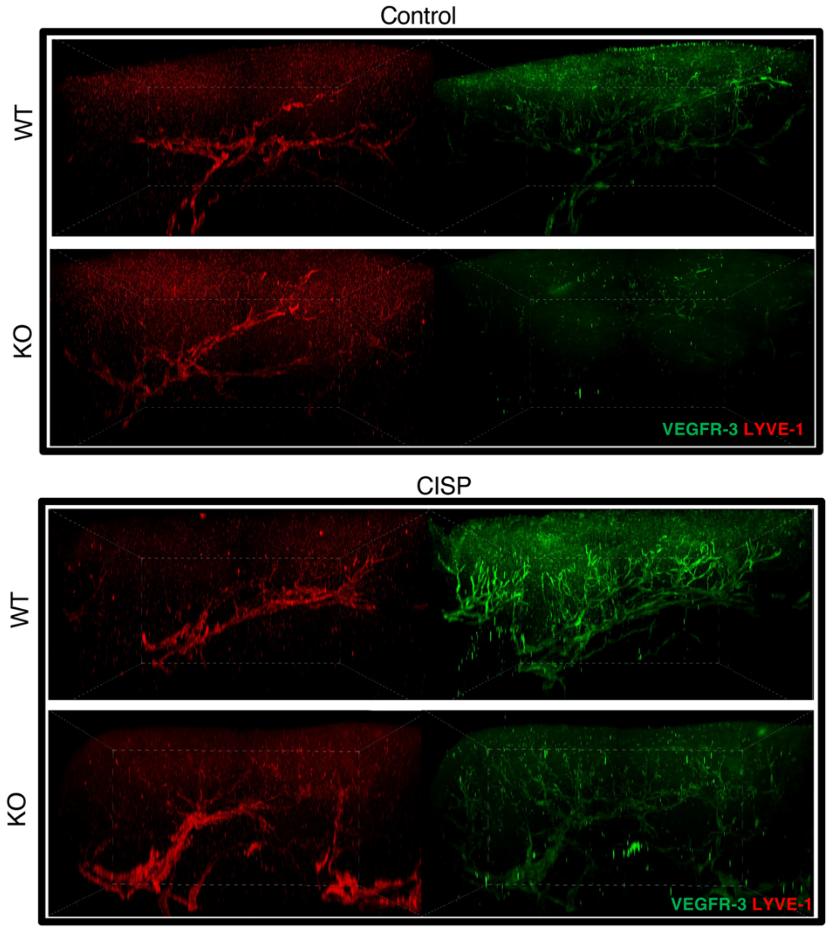

Supplemental Figure 4: Individual channels of VEGFR-3 and LYVE-1 maximum intensity projections of optically cleared and immunolabeled kidneys.

Split-channel immunofluorescence images showing VEGFR-3 (green) and LYVE-1 (red) expression in lymphatic vessels of KO and WT control mice under baseline and cisplatin-induced injury conditions. WT= RelA$^{fl/fl}$, KO= VEGFR-3$^{RelA-/-}$.

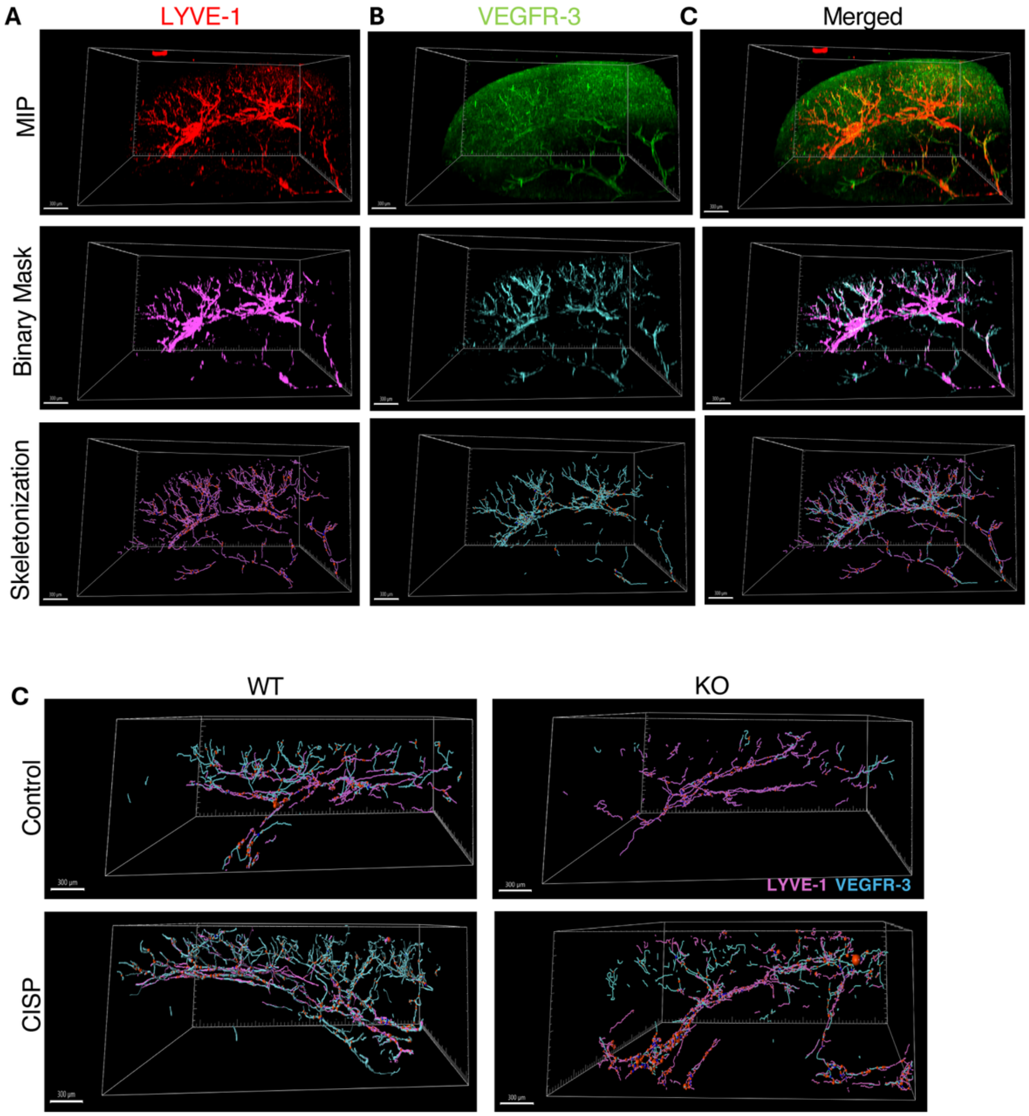

Supplemental Figure 5: Comprehensive image processing workflow for lymphatic vessel quantification using Imaris

Confocal microscopy images (10X magnification) from a cisplatin-treated WT mouse showing (A) LYVE-1 and (B) VEGFR-3 immunolabeled lymphatic vessels. (C) Merged LYVE-1 and VEGFR-3 channels. Images were processed as maximum intensity projections (MIPs) following denoising and deconvolution in the NIS-Elements software. Binary masks were generated using manual absolute thresholding (Imaris Surfaces tool), then subjected to segmentation and

skeletonization analysis (Imaris Filaments module). Colored circles indicate branch points for quantitative assessment of vessel complexity. (D) Representative skeletonization analysis (Imaris Filaments module) showing merged LYVE-1 (pink) and VEGFR-3 (blue) lymphatic vessels derived from binary masks of each channel in control and cisplatin-treated knockout and flox mice. WT= $RelA^{fl/fl}$, KO= VEGFR-$3^{RelA-/-}$.

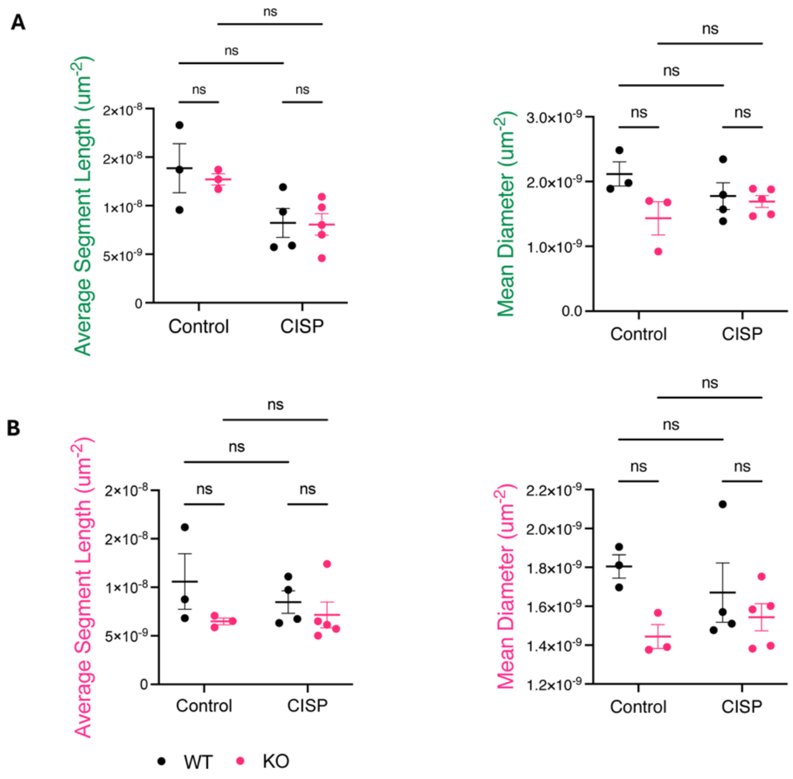

Supplemental Figure 6: VEGFR-3 and LYVE-1 Lymphatic Vasculature Quantification.

(A) Percent changes of VEGFR-3 and (B) LYVE-1 parameters (average segment mean diameter and average segment length) in injured knockout and WT mice. Numbers are represented as raw values divided and normalized by the absolute imaging volume determined from the x- (2038 pixels) y-(2038 pixels) and z- (top um – bottom um) imaging dimensions and pixel size (1.24 um/px). Sample sizes (WT control, KO control, WT cisplatin, KO cisplatin): N=3, 3, 4, 5. WT= RelA$^{fl/fl}$, KO= VEGFR-3$^{RelA-/-}$.

Supplemental Table 1: Primers used in RT-qPCR analysis for murine tissue and hdLECs cell culture.

Supplemental Table 1: Primers used in RT-qPCR analysis for murine tissue and hdLECs cell culture.

Supplemental Table 1

Human primers	Sequence (5' to 3')	Mouse primers	Sequence (5' to 3')
p50 forward	GCAGCACTACTTCTTGACCACC	p50 forward	GAAATTCCTGATCCAGACAAAAAC
p50 reverse	TCTGCTCCTGAGCATTGACGTC	p50 reverse	ATCACTTCAATGGCCTCTGTGTAG
p65 forward	TGAACCGAAACTCTGGCAGCTG	Exon 1 forward	GTTTCCCCTCATCTTTCCCT
p65 reverse	CATCAGCTTGCGAAAAGGAGCC	Exon 3 reverse	GTTCCTGGTCCTGTGTAGC
Lyve1 forward	GCCGACAGTTTGCAGCCTATTG	Lyve 1 forward	GGCTTTGAGACTTGCAGCTATG
Lyve1 reverse	CCGAGTAGGTACTGTCACTGAC	Lyve 1 reverse	GCAGGAGTTAACCCAGGTGT
Prox1 forward	CTGAAGACCTACTTCTCCGACG	Prox1 forward	GAAGGGCTATCACCCAATCA
Prox1 reverse	GATGGCTTGACGTGCGTACTTC	Prox1 reverse	TGAACCACTTGATGAGCTGC
Flt4 forward	TGCGAATACCTGTCCTACGATGC	Flt4 forward	CCATCGAGAGTCTGGACAGC
Flt4 reverse	CTTGTGGATGCCGAAAGCGGAG	Flt4 reverse	CCGGGATGGTGGTCACATAG
Tnfsf15 forward	CACCACATACCTGCTTGTCAGC	Pdpn forward	ACAACCACAGGTGCTACTGGAG
Tnfsf15 reverse	TCTCCGTCTGCTCTAAGAGGTG	Pdpn reverse	GTTGCTGAGGTGGACAGTTCCT
Pdpn forward	GTGCCGAAGATGATGTGGTGAC	p52 forward	CTGGTGGACACATACAGGAAGAC
Pdpn reverse	GGACTGTGCTTTCTGAAGTTGGC	p52 reverse	ATAGGCACTGTCTTCTTTCACCTC
Gapdh forward	GCCAAAAGGGTCATCATCTC	Gapdh forward	ATCATCCCTGCATCCACT
Gapdh reverse	GGCCATCCACAGTCTTCT	Gapdh reverse	ATCCACGACGGACACATT
		Vegfc forward	AGACGGACACACATGGAGGT
		Vegfc reverse	AAAGACTCAATGCATGCCAC
		Ccl21a forward	AAGGCAGTGATGGAGGGGGT
		Ccl21a reverse	CTTAGAGTGCTTCCGGGGTG
		GFP forward	CAGAAGAACGGCATCAAGGTG
		GFP reverse	GGACTGGGTGCTCAGGTAGTG
		Vcam1 forward	GCTATGAGGATGGAAGACTCTGG
		Vcam1 reverse	ACTTGTGCAGCCACCTGAGATC

SUPPLEMENTAL MATERIAL

Supplemental Figures and Videos can be found at: https://figshare.com/s/8c219fc9523b33c88f1d

SUPPLEMENTAL MATERIAL

Supplemental Figures and Videos can be found at: https://figshare.com/s/8c219fc9523b33c88f1d
