# Supplementary figures and images for: NF-κB–driven lymphangiogenesis affects kidney function via a VEGFR-3–mediated pathway
Source: JCI Insight. 2026 Jan 22;11(5):e198992. doi: 10.1172/jci.insight.198992 (PMC13041690; doi:10.1172/jci.insight.198992)

# Full unedited blots for Figure 5A

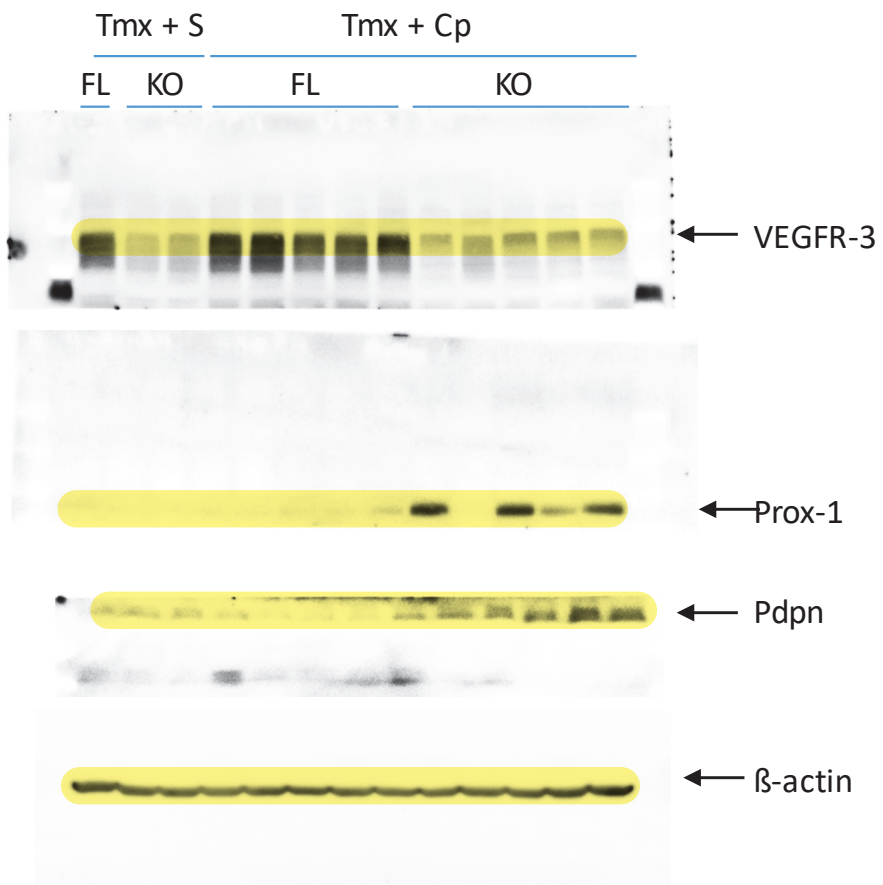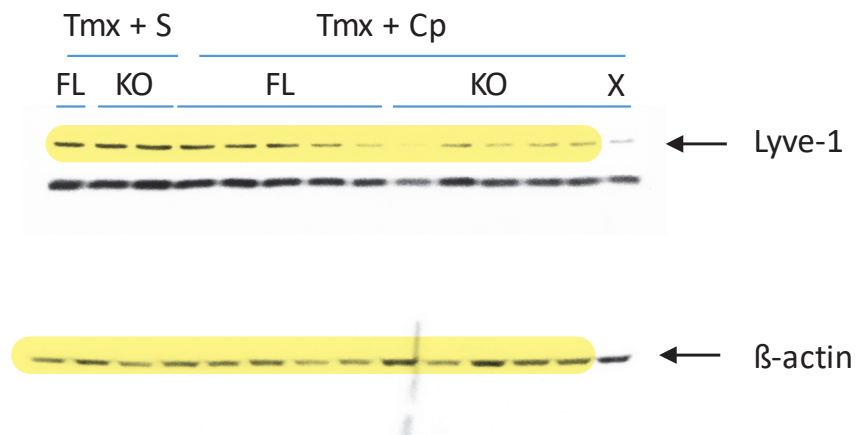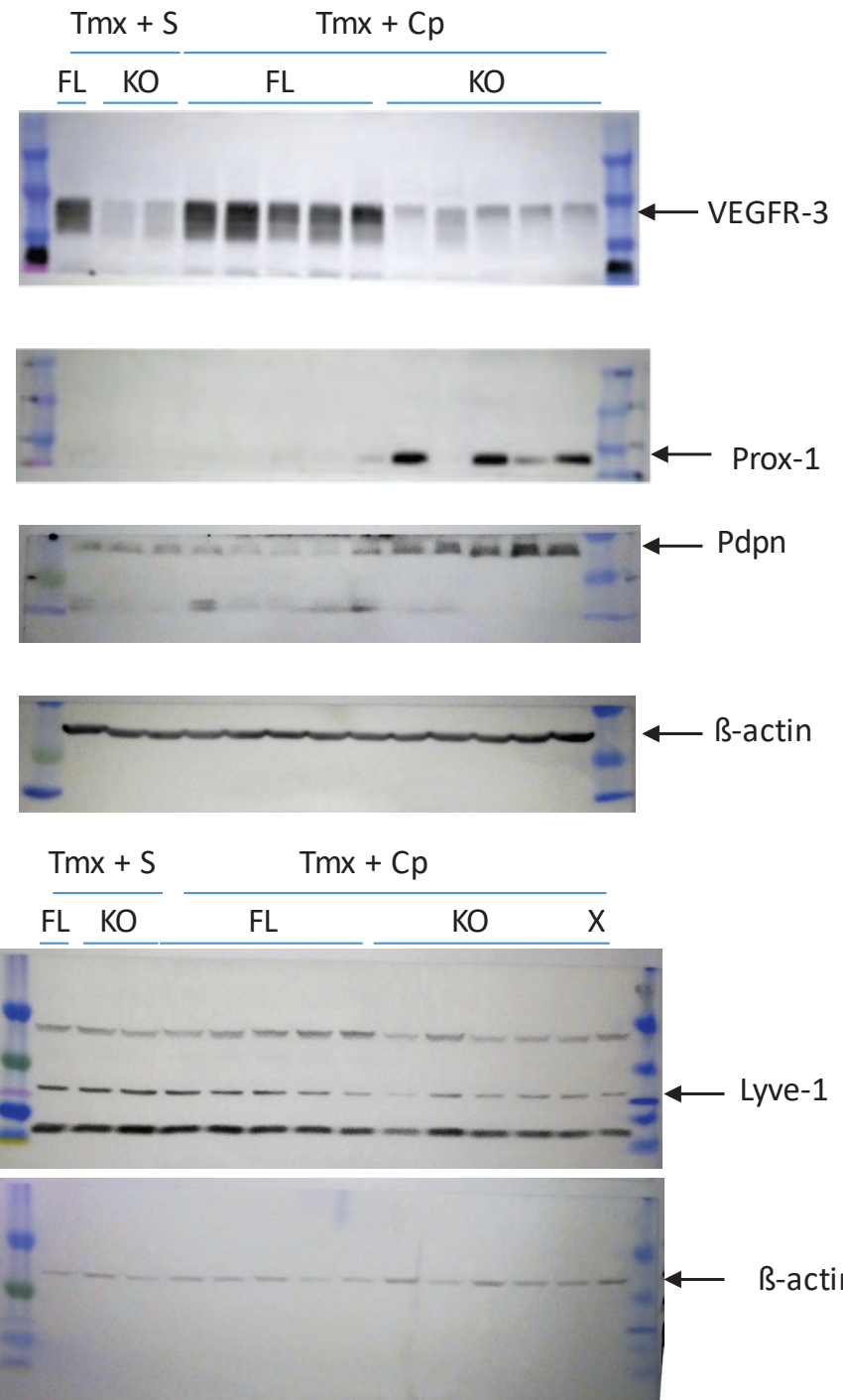

# Full unedited blots for Supp. Figure 1A

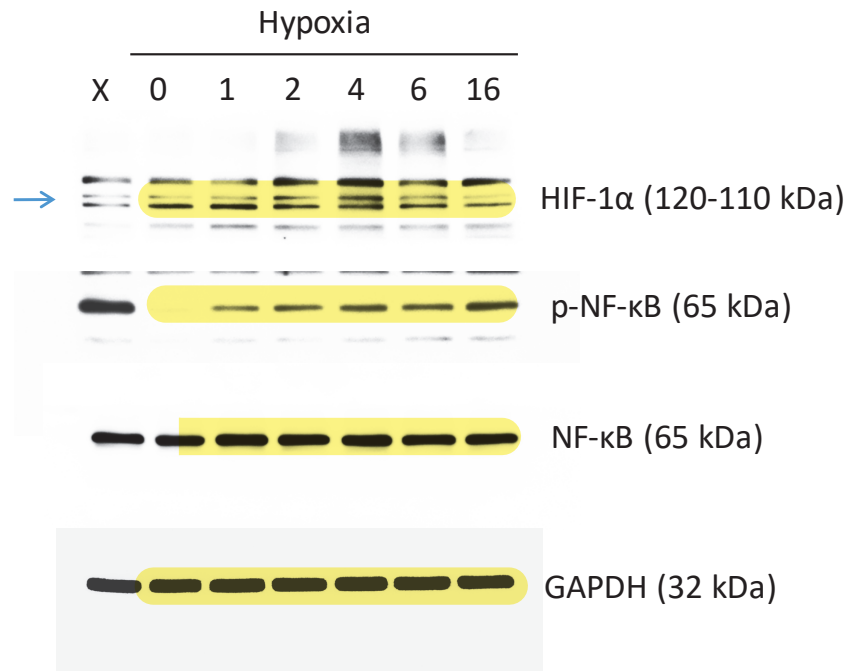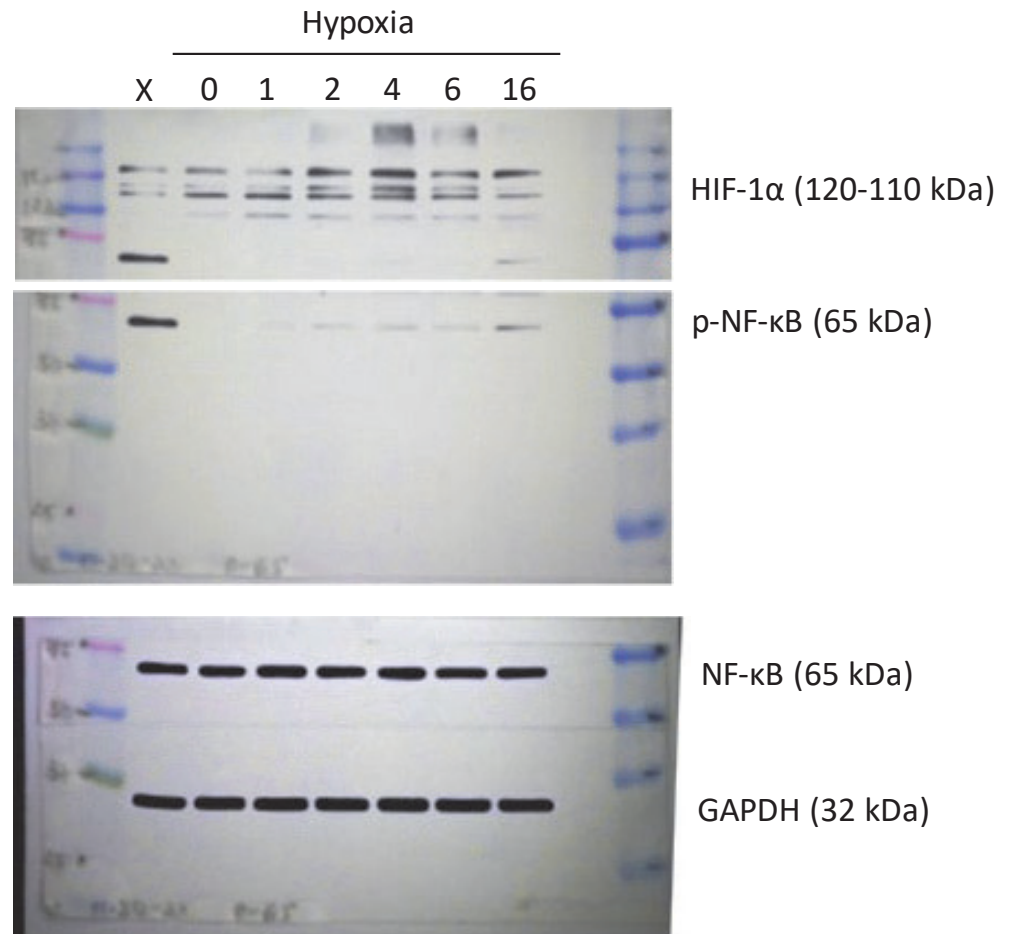

Supplement: Unedited blot and gel images [file jciinsight-11-198992-s192.pdf]
